# Using LASSO Regression to Estimate the Population-Level Impact of Pneumococcal Conjugate Vaccines

**DOI:** 10.1093/aje/kwad061

**Published:** 2023-03-17

**Authors:** Anabelle Wong, Sarah C Kramer, Marco Piccininni, Jessica L Rohmann, Tobias Kurth, Sylvie Escolano, Ulrike Grittner, Matthieu Domenech de Cellès

**Keywords:** counterfactual prediction, epidemiologic methods, LASSO regression, least absolute shrinkage and selection operator, pneumococcal conjugate vaccines, *Streptococcus pneumoniae*, vaccine impact

## Abstract

Pneumococcal conjugate vaccines (PCVs) protect against diseases caused by *Streptococcus pneumoniae*, such as meningitis, bacteremia, and pneumonia. It is challenging to estimate their population-level impact due to the lack of a perfect control population and the subtleness of signals when the endpoint—such as all-cause pneumonia—is nonspecific. Here we present a new approach for estimating the impact of PCVs: using least absolute shrinkage and selection operator (LASSO) regression to select variables in a synthetic control model to predict the counterfactual outcome for vaccine impact inference. We first used a simulation study based on hospitalization data from Mexico (2000–2013) to test the performance of LASSO and established methods, including the synthetic control model with Bayesian variable selection (SC). We found that LASSO achieved accurate and precise estimation, even in complex simulation scenarios where the association between the outcome and all control variables was noncausal. We then applied LASSO to real-world hospitalization data from Chile (2001–2012), Ecuador (2001–2012), Mexico (2000–2013), and the United States (1996–2005), and found that it yielded estimates of vaccine impact similar to SC. The LASSO method is accurate and easily implementable and can be applied to study the impact of PCVs and other vaccines.

## Abbreviations

IPD: invasive pneumococcal diseases
IRR: incidence rate ratio
ITS: interrupted time series
LASSO: least absolute shrinkage and selection operator
PCV: pneumococcal conjugate vaccine
SC: synthetic control with Bayesian variable selection
SF: season-forced
STL + PCA: seasonal-trend decomposition using locally estimated scatterplot smoothing plus principal component analysis
SU: season-unforced

The bacterium *Streptococcus pneumoniae* (pneumococcus) poses a substantial health burden globally. Although it typically colonizes the human nasopharynx asymptomatically, infection can lead to disease ranging from mild (e.g., otitis media) to severe (e.g., pneumonia, meningitis) (1).

Antipneumococcal vaccines were developed to combat pneumococcal infections. The most widely used is pneumococcal conjugate vaccine (PCV), in which several types of pneumococcal capsular polysaccharides are conjugated to carrier proteins to elicit immunity to a subset of approximately 100 serotypes of pneumococcus (2). Following the widespread adoption of a 7-valent PCV (PCV7) in national childhood immunization programs, PCVs of higher valency (e.g., 10-valent (PCV10), 13-valent (PCV13)) were introduced (3, 4). Unlike previous antipneumococcal vaccines, which merely reduced the risk of disease (5), PCVs also protect against carriage of vaccine serotypes and can therefore help to achieve herd immunity (6, 7).

Randomized controlled trials have demonstrated PCV efficacy in disease prevention (8) through comparisons of vaccinated and unvaccinated groups. The efficacy measured in randomized controlled trials is different from the actual vaccine impact on a population level—that is, the reduction of disease burden in a population consisting of vaccinated and unvaccinated individuals in comparison with an otherwise similar but universally unvaccinated population (9)—after PCV introduction. Randomized controlled trials may substantially underestimate vaccine impact on the population level, because vaccinating infants with PCV protects not only the vaccinated but also unvaccinated children and adults against invasive pneumococcal diseases (IPD) and pneumonia (10–12).

Addressing the limitation of randomized controlled trials in vaccine impact estimation means finding a suitable unvaccinated comparison population, which is difficult, if not impossible. Hence, statistical models are routinely employed to emulate the counterfactual disease burden in a hypothetical unvaccinated version of the population (12–18). Since statistical models rely on observational data such as the incidence rates of pneumococcal diseases before and after the introduction of PCVs, they are prone to confounding (such as changes in surveillance systems, reporting, and population demographic characteristics), as acknowledged in previous studies (12, 14, 15, 18). To tackle unmeasured confounding, new methods, consistent with the synthetic control framework (19, 20), compare the trends for pneumococcal diseases with other control conditions that are unaffected by PCVs. One may select controls a priori (17), via Bayesian variable selection (21, 22), or via principal component analysis (23); however, no consensus exists on how best to select controls in order to construct the counterfactual (17, 21–24).

In this study, we present a novel approach for selecting controls under the synthetic control framework for estimation of PCVs’ impact using least absolute shrinkage and selection operator (LASSO) regression, which simultaneously performs variable selection and parameter estimation (25). Through comprehensive simulations, we show that LASSO regression can achieve accurate counterfactual prediction for vaccine impact inference. We further present an application using real-world data to empirically illustrate PCVs’ impact on pneumococcal diseases in different age groups and countries.

## METHODS

### Data

We used monthly hospitalization data originally published and described by Bruhn et al. (21). These data consisted of routinely collected information on reasons for hospitalization in Brazil, Chile, Ecuador, Mexico, and 10 US states, provided either by each individual country’s Ministry of Health or its health-care statistics agency. In this study, we excluded data from Brazil because of a coding shift for the cause of hospitalization in 2008 due to a reimbursement policy change (21). We included all available data periods (which differed by country; see Table 1) for all countries except the United States. For the United States, as in the study by Bruhn et al. (21), we excluded the period 2006–2010 and focused exclusively on the early postvaccine period. In the US data set, counts less than 10 were masked for privacy reasons; therefore, we imputed these values by randomly drawing values between 0 and 9, and we further excluded the period 1994–1995 due to a considerable amount of masked data. The data were aggregated into 8 age groups (<1, 1, 2–4, 5–17, 18–39, 40–64, 65–79, and ≥80 years) for Ecuador, Mexico, and the United States. For Chile, the youngest 2 age groups (<1 year and 1 year) were combined.

**Table 1:** Characteristics of Monthly Hospitalization Count Data Sets for Persons Receiving Pneumococcal Conjugate Vaccine in 4 Countries, 1996–2013^a^

**Country**	**Data Period**	**Type of PCV** ^**b**^	**Date of Introduction** ^**b**^	**Evaluation Period**	**Median No. (Range) of Pneumonia Hospitalizations per Month** ^**c**^	
					**Prevaccine Period**	**Postvaccine Period**
Chile	January 2001–December 2012	PCV10	January 2011	July 2011–December 2012	5,151 (2,586–13,993)	5,036 (2,645–10,520)
Ecuador	January 2001–December 2012	PCV10	January 2010	January 2011–December 2012	1,958 (980–3,658)	2,926 (1,959–5,910)
Mexico	January 2000–December 2013	PCV7	January 2006	January 2010–December 2011	2,246 (1,165–5,375)	3,316 (1,424–6,548)
United States^d^	January 1996–December 2005	PCV7	January 2000	January 2002–December 2004	30,089 (21,517–51,772)	34,517 (25,244–56,984)

Abbreviations: PCV, pneumococcal conjugate vaccine; PCV7, 7-valent pneumococcal conjugate vaccine; PCV10, 10-valent pneumococcal conjugate vaccine.

^a^ For each country, we report the data period included in this study, the type of PCV being introduced, and the date of PCV introduction, as well as how we defined the evaluation period. A full description of the data sets has been published by Bruhn et al. (21).

^b^ Data regarding the type of PCV and the year of introduction were obtained from Bruhn et al. (21), Carnalla-Barajas et al. (47), and the Pan American Health Organization (54).

^c^ Median monthly hospitalization counts (with IQRs) are presented for the outcome, all-cause pneumonia, for all age groups, in the prevaccine and postvaccine periods, to illustrate the scope of the disease burden captured in these data sets.

^d^ The US data came from 10 states: Arizona, Colorado, Iowa, Massachusetts, New Jersey, New York, Oregon, Utah, Washington, and Wisconsin.

### Outcome variable

Our study’s primary endpoint was all-cause pneumonia hospitalization, an established indicator for PCVs’ impact (12, 14, 15, 17, 21, 26). All-cause pneumonia was defined as the presence of *International Classification of Diseases, Tenth Revision*, codes J12–J18 (Chile, Ecuador, and Mexico) or *International Classification of Diseases, Ninth Revision*, codes 480–486 (United States) in the diagnostic field of the electronic hospitalization database (21). In the US data set, we analyzed 4 additional disease endpoints: 1) IPD (pneumococcal meningitis and pneumococcal septicemia); 2) pneumococcal/lobar pneumonia; 3) all-cause pneumonia according to Griffin et al.’s (27) definition (pneumonia listed in the first diagnostic field or listed after a first diagnosis of sepsis, meningitis, or empyema); and 4) all-cause pneumonia with a less specific definition (pneumonia listed in any of the diagnostic fields).

### Control variables and other input variables

Following the method of Bruhn et al. (21), we included the time series of monthly counts of control conditions (“control variables”), which may be associated with all-cause pneumonia but are not themselves affected by PCV (e.g., dermatological conditions, urinary tract infections, etc.). Counts for all control variables were log-transformed and standardized. In addition, we included 11 Fourier functions (6 pairs of sine and cosine functions (with frequency *s*/12) per month (*s* = 1, … 6), minus 1 sine function equal to 0 for *s* = 6) with annual periodicity to model the background seasonality of pneumonia (28, 29) over a period of 12 months (“seasonal variables”). Finally, we included the natural logarithm of nonrespiratory hospitalization as an offset to control for changes in population size or changes in the surveillance system. A complete list of all input variables can be found in Web Appendix 1, Web Table 1 (available at https://doi.org/10.1093/aje/kwad061).

### Data period

For all methods except the interrupted time series (ITS) method, the full data period was divided into 3 parts: 1) the prevaccine period, within which the regression model was trained; 2) the implementation period, during which data were not used; and 3) the evaluation period, during which the outcome incidence rate ratio (IRR) was calculated. The prevaccine period varied by country depending on data availability and the date of vaccine introduction. The implementation period varied depending on the rollout progress of the country. The evaluation period was at least 18 months long in each country. Country-specific data periods are detailed in Table 1, and the fitting period for each method is shown in Table 2.

**Table 2:** Summary of How Comparator Methods Were Implemented in the Simulation Study

**Method**	**Variables Supplied**	**Variable Selection?**	**Period of Fit**	**Method of Calculating Counterfactual**	**Method of Calculating IRR**
ITS	11 seasonal variables, intervention indicator variable	No	Whole period	By predicting postvaccine period outcome after changing the intervention indicator variable from 1 to 0	Fitted outcome/counterfactual outcome
LASSO	11 seasonal variables, control variables	Yes	Prevaccine period	By predicting postvaccine period outcome based on prevaccine fit	Observed outcome/counterfactual outcome
SC	11 seasonal variables, control variables	Yes	Prevaccine period	By predicting postvaccine period outcome based on prevaccine fit	Observed outcome/counterfactual outcome
STL + PCA	11 seasonal variables, control variables	No^a^	Prevaccine period	By predicting postvaccine period outcome based on prevaccine fit	Observed outcome/counterfactual outcome

Abbreviations: IRR, incidence rate ratio; ITS, interrupted time series; LASSO, least absolute shrinkage and selection operator; SC, synthetic control with Bayesian variable selection; STL + PCA seasonal-trend decomposition using locally estimated scatterplot smoothing plus principal component analysis.

^a^ STL + PCA does not select variables but includes a principal component that summarizes the extracted smoothed trends of variables supplied.

### Statistical models

We tested LASSO regression, an extension of generalized linear regression that decreases the variance of regression coefficients and the prediction error by adding a term to the log-likelihood to penalize model complexity (25). This leads to a parsimonious model with a subset of control variables that best predicts the outcome. In our implementation, we tested 2 variants of the LASSO regression model: The first included all seasonal variables by default (season-forced (SF)), while the second treated seasonal variables as control variables and allowed LASSO regression to select from them (season-unforced (SU)); model selection was based on either 10-fold cross-validation or the Akaike information criterion (30). The selected model was refitted onto the entire prevaccine period to predict the counterfactual outcome ($\tilde{Y}_T$) during the evaluation period—that is, the hospitalization counts that would have occurred in the population if PCV had not been introduced, assuming that the distribution and associations of the population features captured in the prevaccine period data remained unchanged. With the LASSO-predicted counterfactual under the no-vaccine scenario and the observed outcome (${Y}_T)$, we calculated the vaccine impact using equation 1. An IRR less than 1 indicates a reduction in all-cause pneumonia hospitalization due to the vaccination program.

(1) \begin{align*} \mathrm{IRR}=\frac{Y_T}{\tilde{Y_T}}=\frac{\sum_{t\in T}{Y}_t}{\sum_{t\in T}\tilde{Y_t}}, \end{align*}

where $T$ is the set of time points during the evaluation period.

We compared LASSO regression with 3 established methods in the field of vaccine impact estimation: namely, ITS, synthetic control with Bayesian variable selection (SC) (21, 22), and seasonal-trend decomposition using locally estimated scatterplot smoothing plus principal component analysis (STL + PCA) (23). All 4 methods are summarized in Table 2 and detailed in Web Appendix 2.

### Performance assessment with simulated data

To assess the performance of all methods in estimating vaccine impact, we designed a simulation study. We generated the outcome, monthly pneumonia hospitalization (${Y}_t$), based on a combination of *n* (*n* = {5, 10}) control variables (${X}_1,{X}_2,\dots, {X}_n$) randomly selected from the list of control variables available in the Mexico data set (21). We then incorporated an intercept ($\mathrm{\alpha}$), the logarithm of nonrespiratory hospitalization (NRH) as the offset ($\ln \left({\mathrm{NRH}}_t\right)$), background seasonality (${S}_t$), and a vaccine impact component ($\mathrm{\gamma}$) into the equation to generate the logarithm of the expected number of monthly pneumonia hospitalizations. A predetermined value was assigned to $\mathrm{\gamma}$ starting from the time point of PCV introduction (${t}_{\mathrm{vac}}$). Assuming a Poisson distribution for the outcome, we simulated 100 time series; thus, the variability of the simulated time series originated from the Poisson variation. The model is represented by equation 2:

(2) $$ \begin{align*} {\displaystyle \begin{array}{c}{Y}_t\sim \mathrm{Poisson}\left({\mathrm{\mu}}_t\right);\\ {}\ln \left({\mathrm{\mu}}_t\right)=\mathrm{\alpha} +\ln \left({\mathrm{NRH}}_t\right)\!+\!\sum \limits_{i=1}^n\ {\mathrm{\beta}}_i\ {X}_{it}+{S}_t+\mathrm{\gamma}1\left(\ t\ge {t}_{\mathrm{vac}}\right)\!, \\ {}\mathrm{where}\ \mathrm{\alpha} =\ln \left(\frac{\overline{Y}}{\overline{\mathrm{NRH}}}\right)\ \mathrm{and}\\ {}\ \hfill{S}_t=\sum \limits_{s=1}^6{\mathrm{\delta}}_s\cos \left(\frac{2\mathrm{\pi} st}{12}\right)+\sum \limits_{s=1}^5{\mathrm{\zeta}}_s\sin \left(\frac{2\mathrm{\pi} st}{12}\right). \hfill(2)\end{array} } \end{align*}$$

The intercept, α, is calculated as the logarithm of the mean ratio of pneumonia hospitalization to all nonrespiratory hospitalization (ln($\overline{Y}/\overline{\mathrm{NRH}}$)). In all simulations, we assumed a vaccine with null impact ($\mathrm{\gamma} =0$), IRR = 1, except in the sensitivity analysis using a nonnull vaccine. The simulated data were screened to ensure they were realistic, such that the maximum ratio of annual maximum to minimum for the expected count of the outcome in any simulation set would not exceed 10. A full description of parameter values is included in Web Appendix 3.

**Figure 1:**
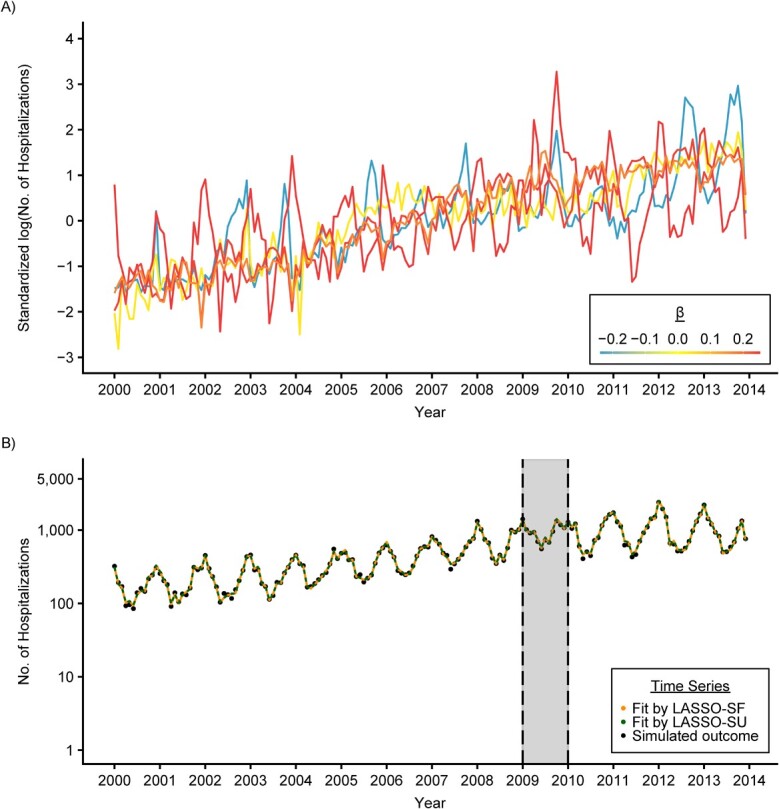
An illustration of how outcome was simulated based on 5 randomly selected control variables in the simulation study. A) Time series of the first set of 5 control variables selected randomly for the simulation of outcome data, colored by the association size, β, assigned to each of them (see Web Appendix 2 for other scenarios that used different sets of 5 control variables to generate the outcome). The 5 control variables shown here are hospitalizations due to 1) health examinations (orange; β = 0.17), 2) nonpneumonia infections (blue; β = −0.23), 3) dermatological conditions (red orange; β = 0.24), 4) nonpneumococcal septicemia (yellow; β = 0.04), and 5) bronchitis and bronchiolitis (red; β = 0.25). B) Time series of the simulated outcome, all-cause pneumonia (black point), and the model prediction from LASSO-SF (orange solid line) and LASSO-SU (green dotted line). The implementation period is marked by the gray area. The *y*-axis is log-transformed. LASSO, least absolute shrinkage and selection operator; SF, season-forced; SU, season-unforced.

We tested the performance of all methods across 4 scenarios. First, we used 5 randomly selected control variables and a seasonal variable to generate the outcome. This analysis was performed 5 times (simulation sets A–E) with resampling of the 5 control variables. To illustrate this process, we plotted the first set of 5 control variables (set A) and their assigned $\mathrm{\beta}$ values used to generate the outcome time series in Figure 1A and the generated outcome time series in Figure 1B. Second, we repeated this analysis with 10 control variables (simulation sets F–J). Third, we tested the performance of all methods on sparse data, which may affect the performance of these methods (23). We generated the sparse data (simulation set K) by taking a 10% binomial subsample from the outcome in set A. A flowchart for the outcome simulation procedures can be found in Web Appendix 3, Web Figure 1; plots similar to Figures 1A and 1B for simulation sets A–E can be found in Web Appendix 3, Web Figure 2. The complete list of control variables used to simulate the outcome can be found in Web Appendix 1, Web Table 1.

Finally, we tested all methods under a fourth scenario (simulation sets L–P), in which the variables causing the outcome were unavailable. We used 3 control variables (C1–C3) to generate 4 conditions (Z1–Z4) and the outcome, such that the conditions Z1–Z4 and the outcome were not causally related but were non–causally associated via common causes. We then removed C1–C3 and their associated control variables (i.e., diagnosis from the same chapter of the *International Classification of Diseases, Tenth Revision*) from the list of control variables. This scenario is more realistic because observed associations between different causes of hospitalization are likely to be due to common causes rather than direct causal influence. A directed acyclic graph depicting the underlying data-generation process under this scenario can be found in Web Appendix 3, Web Figure 3, and the control variables used for outcome simulation are provided in Web Appendix 1, Web Table 1.

To assess the risk of type II error, we performed a sensitivity analysis under the first scenario (sets A–E) by testing LASSO-SF and LASSO-SU for a nonnull, low-impact (1 − IRR = 10%) vaccine.

We evaluated performance by comparing each method’s estimates with the true IRR value. In the LASSO regression and ITS, we report uncertainty of estimation as the 95% prediction intervals of the IRR obtained from each simulation, extracted from the 2.5th and 97.5th percentiles of the Poisson distribution of the predicted values. In SC and STL + PCA, we report the 95% credible intervals for the IRR from each simulation, extracted from the 2.5th and 97.5th percentiles of the Bayesian posterior distributions. In frequentist LASSO methods, the usual statistical constructs such as confidence intervals and *P* values do not exist in the method’s implementation (31, 32); therefore, we report different uncertainty measures for different methods, and they are not directly comparable. For each set of 100 simulations, we measured the accuracy of each method by calculating the mean IRR with its standard deviation. We measured the precision as the width of the 95% uncertainty intervals. For each set of 100 simulations, we assessed performance stability by coverage (proportion of the time that the uncertainty interval contained the true value). For each scenario, we report these performance indicators as a range over the 100 simulations.

### Application to real-world data

In the analysis of the primary endpoint (all-cause pneumonia), LASSO-SF, LASSO-SU, and SC were applied to each age group in each country. Similar to the procedure used on the simulated data, we fitted models to the prevaccine period and used these models to predict the counterfactual outcomes. We performed model selection, prediction of the counterfactuals, and calculation of vaccine impact using the same aforementioned procedures used to assess performance in the simulated data; we then compared the results from the 3 methods. In addition, we explored the use of maximum entropy bootstrapping (33) for the outcome and covariates to calculate approximate 95% confidence intervals for the LASSO estimates in case the 95% prediction intervals became overly narrow. The bootstrap procedures are described in Web Appendix 4, and all of the uncertainty intervals used are summarized in Web Appendix 4, Web Table 2.

We then applied LASSO-SF, LASSO-SU, and SC on the US data using different endpoints (IPD, pneumococcal pneumonia, and 2 definitions of all-cause pneumonia) and compared the results. We performed a sensitivity analysis by removing “bronchitis and bronchiolitis” from the list of possible control variables for LASSO selection, because this control variable could be affected by PCV and violate the assumption that all control variables are not affected by the health intervention (21, 34).

### Numerical implementation

All analyses were conducted in RStudio with R, version 4.1.0 (35). LASSO regression was implemented using the package “glmnet,” version 4.1-2 (36). SC and STL + PCA were implemented using the package “InterventionEvaluatR,” version 0.1 (37). Maximum entropy bootstrapping was implemented using the package “meboot,” version 1.4-9.2 (33). The project’s R dependencies were recorded by the package “renv,” version 0.14.0 (38), for reproducibility.

## RESULTS

### Performance assessment with 5 causal control variables

**Figure 2:**
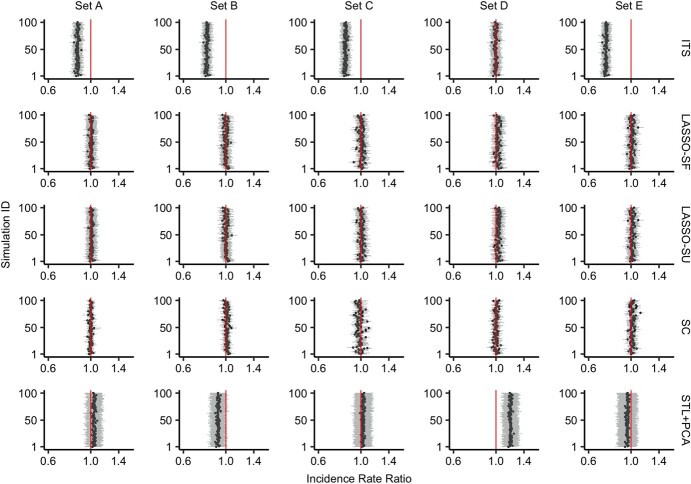
Incidence rate ratios (IRRs) for all-cause pneumonia estimated via various methods for simulations 1–100 in different simulation sets A–E. Each row shows the estimates obtained using a different method (from top to bottom: interrupted time series (ITS); least absolute shrinkage and selection operator, season-forced (LASSO-SF); least absolute shrinkage and selection operator, season-unforced (LASSO-SU); synthetic control with Bayesian variable selection (SC); and seasonal-trend decomposition using locally estimated scatterplot smoothing plus principal component analysis (STL + PCA)). Each column represents a scenario with the outcome simulated with a different set of 5 causal control variables (which remained in the data set) and 1 seasonal variable. The 5 causal control variables used for the simulation set, from left to right, were: set A—health examinations, bronchitis and bronchiolitis, dermatological conditions, nonpneumonia infections, and nonpneumococcal septicemia; set B—nonpneumococcal septicemia, urinary tract infection (UTI), diabetes, stroke, and injury; set C—human immunodeficiency virus (HIV) infection, cholelithiasis, dermatological conditions, endocrinological conditions, and congenital conditions; set D—UTI, dermatological conditions, health examinations, diabetes, and bronchitis and bronchiolitis; and set E—HIV infection, gynecological conditions, gastrointestinal conditions, nonpneumococcal septicemia, and appendicitis. Each panel shows the result from 100 simulations; the points represent the estimated IRR, and the error bars represent the 95% uncertainty interval. The red vertical line indicates the true impact of the vaccine in the simulation, which was 1 in all of our simulation scenarios; here, an IRR higher than 1 means underestimation of the vaccine’s impact and an IRR lower than 1 means overestimation of the vaccine’s impact. All *x*-axes are log-transformed.

Figure 2 shows the vaccine impact estimated by each method for each simulation in 5 scenarios (sets A–E). In each scenario, a different set of 5 randomly selected control variables was used to generate the outcome. LASSO-SF and LASSO-SU performed well in all 5 scenarios; both achieved high coverage (SF: 97%–100%; SU: 96%–100%) and accurate mean IRRs (SF: 1.00–1.02; SU: 1.00–1.03) with good precision (SF: 0.12–0.13; SU: 0.12–0.13), as shown in Figure 2 and Table 2. The estimates obtained by LASSO-SF and LASSO-SU were similar. In general, LASSO regression tended to select the causal variables (Web Figure 4). When comparing cross-validation selection and selection based on the Akaike information criterion, we did not observe different performance in terms of accuracy and precision, but we noticed that cross-validation selection resulted in models with more variables while selection based on the Akaike information criterion led to more parsimonious models (Web Figure 5). The accuracy and precision of LASSO-SF and LASSO-SU remained robust in the sensitivity analysis, where the vaccine impact was 10% (Web Figure 6).

Other methods showed variable performance. ITS yielded accurate and precise estimates with high coverage in one scenario (set D) but the estimates were biased, although precise, in the other scenarios (sets A, B, C, and E), resulting in variable coverage (0%–100%). Similarly, the mean IRR estimated by STL + PCA was biased in some scenarios (sets B and D), causing the coverage to be variable (0%–100%). SC showed relatively high coverage (78%–94%) and accurate mean IRRs (0.99–1.02) with good precision (0.07–0.11). The performance indicators for all methods are summarized in Table 3.

**Table 3:** Performance of 5 Methods for Estimating the Impact of Pneumococcal Conjugate Vaccine Under Different Simulation Scenarios^a^

**Method**	**Range of Mean IRRs(Range of SDs)**	**Range of Mean Widthsof 95% UIs**	**Range ofCoverage, %**
Simulation sets A–E^b^			
ITS	0.74–1.00 (0.010–0.015)	0.08–0.12	0–100
LASSO-SF	1.00–1.02 (0.015–0.026)	0.12–0.13	97–100
LASSO-SU	1.00–1.03 (0.014–0.023)	0.12–0.13	96–100
SC	0.99–1.02 (0.014–0.035)	0.07–0.11	78–94
STL + PCA	0.91–1.19 (0.012–0.015)	0.17–0.23	0–100
Simulation sets F–J^c^			
ITS	0.75–1.15 (0.009–0.017)	0.09–0.16	0–0
LASSO-SF	0.99–1.03 (0.020–0.028)	0.14–0.14	96–100
LASSO-SU	0.99–1.03 (0.020–0.027)	0.14–0.14	91–100
SC	0.99–1.01 (0.023–0.040)	0.08–0.13	87–95
STL + PCA	0.87–1.23 (0.010–0.017)	0.19–0.23	0–100
Simulation set K^d^^,^^e^			
ITS	0.86 (0.041)	0.31	74
LASSO-SF	1.02 (0.044)	0.40	100
LASSO-SU	1.03 (0.042)	0.41	100
SC	1.02 (0.046)	0.23	98
STL + PCA	1.03 (0.045)	0.25	97
Simulation sets L–P^f^			
ITS	0.75–0.99 (0.011–0.014)	0.08–0.12	0–100
LASSO-SF	0.96–1.03 (0.021–0.027)	0.11–0.13	79–100
LASSO-SU	0.96–1.04 (0.019–0.030)	0.11–0.13	81–100
SC	1.00–1.02 (0.012–0.036)	0.06–0.11	78–97
STL + PCA	0.91–1.08 (0.011–0.014)	0.15–0.24	16–100

Abbreviations: IRR, incidence rate ratio; ITS, interrupted time series; LASSO, least absolute shrinkage and selection operator; SC, synthetic control with Bayesian variable selection; SD, standard deviation; SF, season-forced; STL + PCA seasonal-trend decomposition using locally estimated scatterplot smoothing plus principal component analysis; SU, season-unforced; UI, uncertainty interval.

^a^ We applied 5 methods (ITS, LASSO-SF, LASSO-SU, SC, and STL + PCA) to 16 sets of 100 simulations. We summarized the IRR estimated for each of the 100 simulations using the mean IRR with an SD. We summarized the precision of the IRR estimated for each of the 100 simulations using the mean width of the 95% UI. We report the range of mean IRRs and the range of SDs, as well as the range of the mean widths or the 95% UIs, for each type of simulation scenario, except for set K, where a value is reported instead of a range.

^b^ Simulation sets A–E: outcome was simulated using 5 causal control variables.

^c^ Simulation sets F–J: outcome was simulated using 10 causal control variables.

^d^ Simulation set K: outcome was simulated using the 5 causal control variables as in set A, but the outcome count was 10% as sparse as that in set A.

^e^ Because there was only 1 set of simulations for the sparse outcome (set K), we report the mean IRR with its SD, the mean width of the 95% UI, and coverage, instead of a range.

^f^ Simulation sets L–P: outcome was simulated using 3 causal control variables, which were then removed.

### Performance assessment with 10 causal control variables

The performance of LASSO-SF and LASSO-SU remained robust in another 5 scenarios (sets F–J), in which different sets of 10 randomly selected control variables were used to generate the outcome (Web Figure 7). As the number of causal control variables increased from 5 to 10, the number of control variables that were consistently selected by LASSO-SF and LASSO-SU also increased (Web Figure 8). Again, the performance of SC was satisfactory and consistent, while that of the other methods appeared to be variable (Web Figure 7). The performance is summarized in Table 3.

### Performance assessment with sparse data

When the monthly hospitalization counts became as sparse as 10% of the simulated outcome in set A (set K), the performance of all methods remained consistent in terms of accuracy, but the precision notably decreased as the 95% uncertainty intervals for the IRR estimated by all methods widened considerably, which in turn increased coverage (Web Figure 7).

### Performance assessment with noncausal control variables

**Figure 3:**
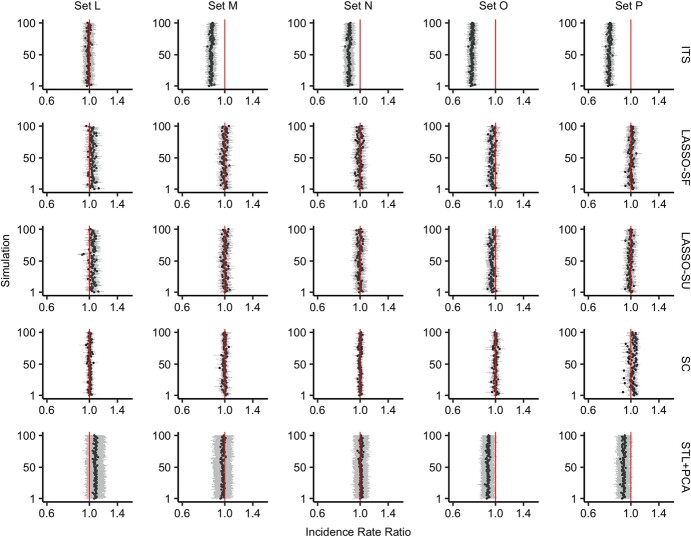
Incidence rate ratios (IRRs) for all-cause pneumonia estimated via various methods for simulations 1–100 in simulation sets L–P. Each row shows the estimates obtained using a different method (from top to bottom: interrupted time series (ITS); least absolute shrinkage and selection operator, season-forced (LASSO-SF); least absolute shrinkage and selection operator, season-unforced (LASSO-SU); synthetic control with Bayesian variable selection (SC); and seasonal-trend decomposition using locally estimated scatterplot smoothing plus principal component analysis (STL + PCA)). Each column represents a scenario with the outcome simulated from a different set of 3 causal control variables (which were then removed alongside other control variables under the same chapter of the *International Classification of Diseases, Tenth Revision*, leaving behind only noncausal control variables) and 1 seasonal variable. The 3 causal control variables used for the simulation set, from left to right, were: set L—neoplasms, urinary tract infection (UTI), and bronchitis and bronchiolitis; set M—neoplasms, UTI, and diabetes; set N—health examinations, UTI, and bronchitis and bronchiolitis; set O—health examinations, UTI, and diabetes; and set P—gastrointestinal conditions, UTI, and diabetes. Each panel shows the result from 100 simulations; the points represent the estimated IRR, and the error bars represent the 95% uncertainty interval. The red vertical line indicates the true impact of the vaccine in the simulation, which was 1 in all of our simulation scenarios; here, an IRR higher than 1 means underestimation of the vaccine’s impact and an IRR lower than 1 means overestimation of the vaccine’s impact. All *x*-axes are log-transformed.

When tested on the data generated from 3 causal control variables (C1–C3) that were subsequently removed (sets L–P), the estimation by LASSO-SF and LASSO-SU remained accurate and precise (Figure 3, Table 3). In the absence of C1–C3, LASSO-SF and LASSO-SU selected the control variables that were associated with the outcome via the common causes, such as Z2, Z3, or Z4. We also observed that LASSO-SF and LASSO-SU preferentially selected the control variable that was more strongly associated with the common cause, Z2, whereas Z1, the control variable with a weaker association with the common cause, was almost never selected in all 5 scenarios (Web Figure 9).

### Application to real-world data

**Figure 4:**
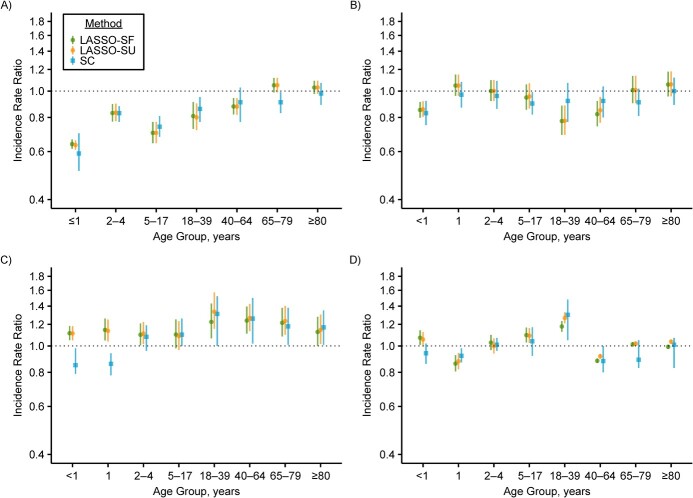
Age-group–specific incidence rate ratios (IRRs) for all-cause pneumonia in 4 countries, estimated via 2 different LASSO methods (least absolute shrinkage and selection operator, season-forced (LASSO-SF) and least absolute shrinkage and selection operator, season-unforced (LASSO-SU)) and synthetic control with Bayesian variable selection (SC). Each panel shows the IRR for all-cause pneumonia in a population whose infants were vaccinated with pneumococcal conjugate vaccine (PCV) as compared with a counterfactual population in which PCV was never introduced, estimated by LASSO-SF (green), LASSO-SU (orange), and SC (blue). The 4 countries are Chile (2001–2012) (A), Ecuador (2001–2012) (B), Mexico (2000–2013) (C), and the United States (1996–2005) (D). The 95% prediction intervals of IRR estimates made via LASSO-SF and LASSO-SU are shown by error bars joined at a circle; the 95% credible intervals of estimates made via SC are shown by error bars joined at a square. Ninety-five percent prediction intervals and 95% credible intervals are different measures of uncertainty, and they are not directly comparable. All *y*-axes are log-transformed.

The characteristics of the 4 countries’ data sets are summarized in Table 1. The IRRs estimated by LASSO-SF and LASSO-SU were comparable to those obtained by SC for Chile, Ecuador, Mexico, and the United States. The 3 methods generally arrived at the same conclusion as to whether there was a significant impact of PCV, except for 2 instances: 1) The 2 LASSO methods found a significant impact of PCV in the age group 40–64 years in Chile, in the age group 18–64 years in Ecuador, and in the age group 40–64 years in the United States, while SC did not; and 2) the SC method detected a significant impact of PCV in the age group 65–79 years in Chile and in the age group <2 years in Mexico, which was not detected by the LASSO methods (see Figure 4). In general, we found that LASSO delivered estimates comparable to those of SC, which has been shown to be a reliable method for vaccine impact estimation (21, 22). While the 95% prediction intervals for LASSO estimates were much narrower than the 95% credible intervals for the SC estimates in the US adult age groups, the 95% confidence intervals for LASSO estimates obtained using maximum entropy bootstrapping (33) were more comparable to the 95% credible intervals for the SC estimates across age groups in all countries (Web Figure 10).

The results using Ecuador, Mexico, and US data were sensitive to removal of “bronchitis and bronchiolitis” from the list of control variables that LASSO regression and SC could select (Web Appendix 5, Web Figure 11).

**Figure 5:**
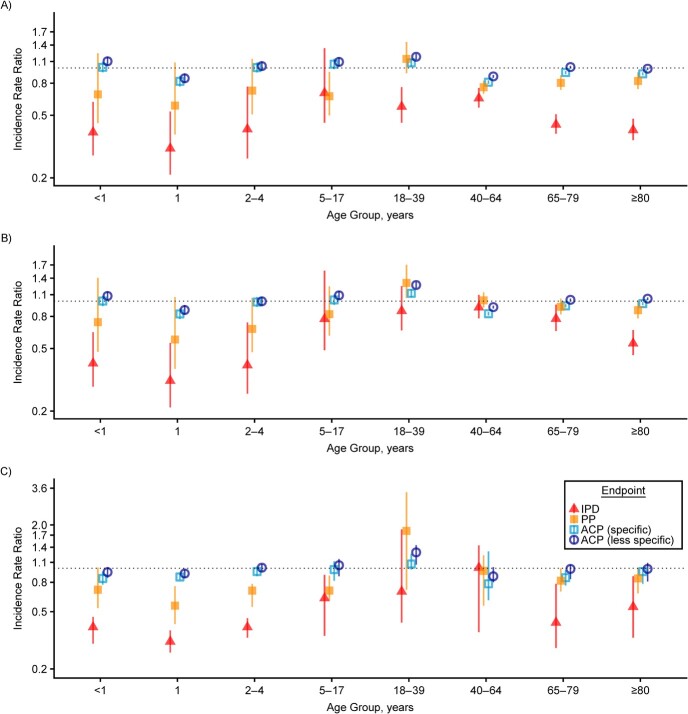
Age-group–specific incidence rate ratios (IRRs) for all-cause pneumonia regarding invasive pneumococcal diseases and other disease endpoints in the United States, estimated via 2 LASSO methods (least absolute shrinkage and selection operator, season-forced (LASSO-SF) and least absolute shrinkage and selection operator, season-unforced (LASSO-SU)) and synthetic control with Bayesian variable selection (SC). Each panel shows the IRRs for 4 disease endpoints in a population whose infants were vaccinated with pneumococcal conjugate vaccine (PCV) as compared with a counterfactual population in which PCV was never introduced, estimated by LASSO-SF (A), LASSO-SU (B), and SC (C). The 4 endpoints used were invasive pneumococcal diseases (IPD; red filled triangles), pneumococcal pneumonia (PP; orange filled squares), all-cause pneumonia (ACP) as the primary diagnosis or as the first diagnosis made after sepsis, meningitis, or empyema (specific; light blue open squares), and ACP as the primary or nonprimary diagnosis (less specific; dark blue open circles). The error bars in panels A and B represent the 95% prediction intervals of the IRR estimates, and the error bars in panel C represent the 95% credible intervals. Ninety-five percent prediction intervals and 95% credible intervals are different measures of uncertainty, and they are not directly comparable. All *y*-axes are log-transformed.

Finally, we applied LASSO-SF, LASSO-SU, and SC to US IPD hospitalization data and compared results for 4 different endpoints: IPD, pneumococcal pneumonia, all-cause pneumonia with a more specific definition, and all-cause pneumonia with a more inclusive definition. LASSO-SF found a statistically significant reduction in IPD hospitalization across all age groups, except for the age group 5–17 years. LASSO-SU and SC found a significant reduction in IPD hospitalization in age groups younger than 5 years and older than 64 years. As the endpoint definition became less specific, the point estimate for reduction became smaller in size, and the 95% uncertainty interval more often included 1 (see Figure 5).

## DISCUSSION

In this study, we aimed to assess whether LASSO regression models can accurately estimate vaccine impact. Using a simulation study, we first assessed the performance of LASSO regression as compared with other commonly implemented methods, including ITS, SC (21, 22), and STL + PCA (23). Upon applying LASSO regression and SC (21, 22) to real-world data, overall we found that LASSO regression allowed for accurate and precise estimation of vaccine impact and performed comparably to established methods, such as SC (21, 22).

The results from the simulation study showed that LASSO regression was able to estimate the predetermined vaccine impact accurately and precisely, and its performance remained stable even under more complex scenarios, such as the ones without any causal variables. While ITS was able to estimate the predetermined vaccine impact accurately in some simulation scenarios, its performance was not robust across scenarios. Because ITS did not include any control variables (only the offset and seasonal terms were included), its assumption that the characteristics in the population remained unchanged throughout the study period limited its performance (39). In practice, control variables can be included in more advanced ITS models to improve performance (12, 39); however, the process of hand-picking control variables is subjective and can introduce biases into the analysis (40). By design, ITS assumes a linear (or exponential, when a log-link function is used) trend for the continuous effect of an intervention (21, 39, 41), which can limit validity because the nature of the persistent effect of an intervention is often unknown or difficult to ascertain.

We compared LASSO regression with 2 other methods—SC (21, 22) and STL + PCA (23). In contrast to a priori selection of control conditions (17), these methods use data-driven approaches to select various control conditions to generate the counterfactual comparator. Therefore, even when confounders are unknown or when the network of causal pathways is complicated, these methods can still help reduce confounding of the causal relationship of interest (17, 21, 23). The results from the simulation study showed stable performance of LASSO methods and SC, while the performance of STL + PCA was not consistent. One possible explanation for the biased estimation by STL + PCA in some of the simulation scenarios is that only 1 principal component was used for the counterfactual prediction, which may not have been sufficient in some scenarios. Our simulation results showed that LASSO regression tended to select the causal variables, or associated variables when no causal variables were available, consistent with its known feature of identifying few predictors with strong associations (30, 42).

When we applied LASSO regression to the real-world data, we found that PCV was associated with reductions in all-cause pneumonia hospitalization in the youngest age groups and in adult age groups (ages 18–64 years) in Chile and Ecuador, as in existing literature (34, 43–45), but we did not observe similar results in Mexico or the United States, which contrasts with published evidence (27, 45, 46). Part of these discrepancies may be explained by the vaccine rollout timeline and coverage. In Mexico, PCV7 was first given to children in resource-poor regions before its official introduction in 2008 (47), and initial universal vaccination in Mexico covered only 2 (instead of 3) doses due to financial constraints (47). In the United States, initial low PCV 3-dose coverage during 2002–2004 may have been insufficient to reach herd immunity in the older age groups (48).

Our results showed that applying LASSO regression to pneumonia hospitalization data was sensitive to removing “bronchitis and bronchiolitis” from the pool of control variables subject to selection by LASSO. One possible explanation for this observation is a potential violation of our assumption that bronchitis and bronchiolitis hospitalization was not affected by PCV. Bruhn et al. (21) and Jimbo Sotomayor et al. (34) highlighted that including bronchitis and bronchiolitis hospitalization can be important for accurate prediction of pneumonia hospitalization due to its association with respiratory syncytial virus infections. Notably, the fraction of bronchitis and bronchiolitis hospitalization caused by pneumococcus and the prevalence of respiratory syncytial virus differ by age group (49–51); therefore, the pathogen-pathogen interactions are potentially different in different age groups.

There were a few limitations in our study that should be considered. First, the simulated data were generated based on time series of 10 or fewer causes of hospitalization, and LASSO tends to perform well in situations where a few variables predict the outcome well because of its property of eliminating variables by shrinking their coefficients to 0. Therefore, the simulation scenarios in our study may have favored LASSO regression. Nevertheless, it is possible that pneumonia hospitalization can be predicted by a few control conditions given its seasonality and relatively well-established etiology. Second, due to privacy-related masking of low monthly case counts in the United States, we imputed these masked values by randomly selecting a number between 0 and 9. Although we do not think this approach posed problems for the primary endpoint analysis because pneumonia hospitalization case counts across all age groups were high (on the scale of 100–10,000), more specific endpoints such as IPD and pneumococcal pneumonia had lower hospitalization counts in younger age groups. However, information from the trend of the time series was retained because the masked value had a definite range (less than 10). Third, using the predicted counterfactual based on prevaccine period data to infer vaccine impact assumes that the relationship between the control conditions and the outcome remained the same before and after PCV introduction. Therefore, if the relationship between the control conditions and the outcome changed around the time of vaccine introduction, the prediction performance of LASSO would have been affected. However, we believe it is unlikely that the relationship between all of the control conditions and the outcome would have been altered at the same time. An exception may be a situation in which a vaccine is introduced to mitigate the effects of a disease that has a very strong impact on lifestyle, mortality, and health-care system capacity, as was seen, for example, during the coronavirus disease 2019 (COVID-19) pandemic (52, 53).

Lastly, a potential limitation of LASSO is the absence of simple methods for quantifying parametric uncertainty, expressed as a confidence interval. In our analyses, we instead calculated the prediction interval, which quantifies only the prediction uncertainty from the Poisson distribution. Nevertheless, these prediction intervals had reasonable coverage across a range of scenarios in our simulations, and were generally comparable to the credible intervals for the SC estimates obtained in the real-word data analyses. A noticeable exception was the US data set, where the prediction intervals appeared too narrow in some age groups with high average case counts. This phenomenon is expected from the properties of the Poisson distribution and the definition of the IRR. Specifically, defining $Y\sim \mathrm{Poisson}\left(\mathrm{\mu} \right)$, where $\mathrm{\mu}$ is the mean of the outcome time series, the IRR can be approximated by $\mathrm{\mu} /Y$, whose standard deviation—calculated using the delta method—is roughly equal to $1/\surd \mathrm{\mu}$ (this scaling function was verified using the empirical data sets). Because of this property, the prediction interval may become too narrow in data sets with large average case counts, and in such cases, we recommend calculating the confidence interval using a bootstrapping approach (such as maximum entropy bootstrapping (33)).

In conclusion, our study offers a comprehensive simulation framework for comparing different methods of estimating vaccine impact and presents a novel approach for counterfactual prediction to infer vaccine impact. Given its stable performance and ease of implementation, we argue that LASSO regression is useful for assessing the impact of other vaccines and ultimately can help analyze epidemiologic data for health policy-making.

## Supplementary Material

Web_Material_kwad061

## References

[1] Weiser JN , Ferreira DM, Paton JC. *Streptococcus pneumoniae*: transmission, colonization and invasion. *Nat Rev Microbiol*. 2018;16(6):355–367. 29599457 10.1038/s41579-018-0001-8 PMC5949087

[2] Ganaie F , Saad JS, McGee L, et al. A new pneumococcal capsule type, 10D, is the 100th serotype and has a large *cps* fragment from an oral streptococcus. *MBio*. 2020;11(3):1–15. 10.1128/mBio.00937-20 PMC7240158 32430472

[3] Tin Tin Htar M , Christopoulou D, Schmitt HJ. Pneumococcal serotype evolution in Western Europe. *BMC Infect Dis*. 2015;15(1):419. 26468008 10.1186/s12879-015-1147-x PMC4606906

[4] Lee LH , Gu XX, Nahm MH. Towards new broader spectrum pneumococcal vaccines: the future of pneumococcal disease prevention. *Vaccine*. 2014;2(1):112–128. 10.3390/vaccines2010112 PMC4494192 26344470

[5] Pletz MW , Maus U, Krug N, et al. Pneumococcal vaccines: mechanism of action, impact on epidemiology and adaption of the species. *Int J Antimicrob Agents*. 2008;32(3):199–206. 18378430 10.1016/j.ijantimicag.2008.01.021

[6] Shiri T , Datta S, Madan J, et al. Indirect effects of childhood pneumococcal conjugate vaccination on invasive pneumococcal disease: a systematic review and meta-analysis. *Global Health*. 2017;5(1):e51–e59. 27955789 10.1016/S2214-109X(16)30306-0

[7] Davis SM , Deloria-Knoll M, Kassa HT, et al. Impact of pneumococcal conjugate vaccines on nasopharyngeal carriage and invasive disease among unvaccinated people: review of evidence on indirect effects. *Vaccine*. 2013;32(1):133–145. 23684824 10.1016/j.vaccine.2013.05.005

[8] Klugman KP , Black S, Dagan R, et al. Pneumococcal conjugate vaccine and pneumococcal common protein vaccines. In: Plotkin SA, Orenstein WA, Offit PA, et al., eds. *Vaccines*. 7th ed. Philadelphia, PA: Elsevier BV; 2013:504–541.

[9] Halloran ME , Longini IM, Struchiner CJ. Design and Analysis of Vaccine Studies. New York, NY: Springer New York; 2010.

[10] European Centre for Disease Prevention and Control . Factsheet about pneumococcal disease. https://www.ecdc.europa.eu/en/pneumococcal-disease/facts. Published August 11, 2020. Accessed November 28, 2020.

[11] Isaacman DJ , McIntosh ED, Reinert RR. Burden of invasive pneumococcal disease and serotype distribution among *Streptococcus pneumoniae* isolates in young children in Europe: impact of the 7-valent pneumococcal conjugate vaccine and considerations for future conjugate vaccines. *Int J Infect Dis*. 2010;14(3):e197–e209. 19700359 10.1016/j.ijid.2009.05.010

[12] Grijalva CG , Nuorti JP, Arbogast PG, et al. Decline in pneumonia admissions after routine childhood immunisation with pneumococcal conjugate vaccine in the USA: a time-series analysis. *Lancet*. 2007;369(9568):1179–1186. 17416262 10.1016/S0140-6736(07)60564-9

[13] Simonsen L , Taylor RJ, Schuck-Paim C, et al. Effect of 13-valent pneumococcal conjugate vaccine on admissions to hospital 2 years after its introduction in the USA: a time series analysis. *Lancet Respir Med*. 2014;2(5):387–394. 24815804 10.1016/S2213-2600(14)70032-3

[14] Berglund A , Ekelund M, Fletcher MA, et al. All-cause pneumonia hospitalizations in children <2 years old in Sweden, 1998 to 2012: impact of pneumococcal conjugate vaccine introduction. *PLoS One*. 2014;9(11):e112211. 25379659 10.1371/journal.pone.0112211 PMC4224441

[15] Nair H , Watts AT, Williams LJ, et al. Pneumonia hospitalisations in Scotland following the introduction of pneumococcal conjugate vaccination in young children. *BMC Infect Dis*. 2016;16(1):390. 27506837 10.1186/s12879-016-1693-x PMC4977871

[16] Alari A , Chaussade H, Domenech De Cellès M, et al. Impact of pneumococcal conjugate vaccines on pneumococcal meningitis cases in France between 2001 and 2014: a time series analysis. *BMC Med*. 2016;14(1):1–11. 27998266 10.1186/s12916-016-0755-7 PMC5175381

[17] Thorrington D , Andrews N, Stowe J, et al. Elucidating the impact of the pneumococcal conjugate vaccine programme on pneumonia, sepsis and otitis media hospital admissions in England using a composite control. *BMC Med*. 2018;16(1):13–14. 29415741 10.1186/s12916-018-1004-z PMC5804014

[18] Hammitt LL , Etyang AO, Morpeth SC, et al. Effect of ten-valent pneumococcal conjugate vaccine on invasive pneumococcal disease and nasopharyngeal carriage in Kenya: a longitudinal surveillance study. *Lancet*. 2019;393(10186):2146–2154. 31000194 10.1016/S0140-6736(18)33005-8 PMC6548991

[19] Abadie A , Diamond A, Hainmueller AJ. Synthetic control methods for comparative case studies: estimating the effect of California’s tobacco control program. *J Am Stat Assoc*. 2010;105(490):493–505.

[20] Brodersen KH , Gallusser F, Koehler J, et al. Inferring causal impact using Bayesian structural time-series models. *Ann Appl Stat*. 2015;9(1):247–274.

[21] Bruhn CAW , Hetterich S, Schuck-Paim C, et al. Estimating the population-level impact of vaccines using synthetic controls. *Proc Natl Acad Sci U S A*. 2017;114(7):1524–1529. 28154145 10.1073/pnas.1612833114 PMC5321019

[22] Kleynhans J , Tempia S, Shioda K, et al. Estimated impact of the pneumococcal conjugate vaccine on pneumonia mortality in South Africa, 1999 through 2016: an ecological modelling study. *PLoS Med*. 2021;18(2):e1003537–e1003515. 33591995 10.1371/journal.pmed.1003537 PMC7924778

[23] Shioda K , Schuck-Paim C, Taylor RJ, et al. Challenges in estimating the impact of vaccination with sparse data. *Epidemiology*. 2019;30(1):61–68. 30334918 10.1097/EDE.0000000000000938 PMC6276862

[24] Schuck-Paim C , Taylor RJ, Simonsen L, et al. Challenges to estimating vaccine impact using hospitalization data. *Vaccine*. 2017;35(1):118–124. 27899227 10.1016/j.vaccine.2016.11.030 PMC5664940

[25] Tibshirani R . Regression shrinkage and selection via the lasso. *J R Stat Soc Ser B*. 1996;58(1):267–288.

[26] Andrade AL , Afonso ET, Minamisava R, et al. Direct and indirect impact of 10-valent pneumococcal conjugate vaccine introduction on pneumonia hospitalizations and economic burden in all age-groups in Brazil: a time-series analysis. *PLoS One*. 2017;12(11):1–19. 10.1371/journal.pone.0184204 PMC5589174 28880953

[27] Griffin MR , Zhu Y, Moore MR, et al. U.S. hospitalizations for pneumonia after a decade of pneumococcal vaccination. *N Engl J Med*. 2013;369(2):155–163. 23841730 10.1056/NEJMoa1209165 PMC4877190

[28] Lin HC , Lin CC, Chen CS, et al. Seasonality of pneumonia admissions and its association with climate: an eight-year nationwide population-based study. *Chronobiol Int*. 2009;26(8):1647–1659. 20030547 10.3109/07420520903520673

[29] Murdoch KM , Mitra B, Lambert S, et al. What is the seasonal distribution of community acquired pneumonia over time? A systematic review. *Australas Emerg Nurs J*. 2014;17(1):30–42. 24507181 10.1016/j.aenj.2013.12.002

[30] James G , Witten D, Hastie T, et al. An Introduction to Statistical Learning. New York, NY: Springer New York; 2013.

[31] Lockhart R , Taylor J, Tibshirani RJ, et al. A significance test for the lasso. *Ann Stat*. 2014;42(2):413–468. 25574062 10.1214/13-AOS1175 PMC4285373

[32] Kyung M , Gilly J, Ghoshz M, et al. Penalized regression, standard errors, and Bayesian lassos. *Bayesian Anal*. 2010;5(2):369–412.

[33] Vinod HD , López-de-Lacalle J. Maximum entropy bootstrap for time series: the meboot R package. *J Stat Softw*. 2009;29(5):1–19.

[34] Jimbo Sotomayor R , Toscano CM, Sánchez Choez X, et al. Impact of pneumococcal conjugate vaccine on pneumonia hospitalization and mortality in children and elderly in Ecuador: time series analyses. *Vaccine*. 2020;38(45):7033–7039. 32981782 10.1016/j.vaccine.2020.09.032

[35] R Core Team . The R Project for Statistical Computing. https://www.r-project.org/. Published May 19, 2021. Accessed August 11, 2021.

[36] Friedman J , Hastie T, Tibshirani R, et al. glmnet: Lasso and Elastic-Net Regularized Generalized Linear Models. https://cran.r-project.org/web/packages/glmnet/index.html. Published June 24, 2021. Accessed August 11, 2021.

[37] Weinberger D . InterventionEvaluatR. https://github.com/weinbergerlab/InterventionEvaluatR.git. Published February 5, 2019. Accessed December 9, 2020.

[38] Ushey K. renv: Project Environments. https://cran.r-project.org/web/packages/renv/index.html. Published July 21, 2021. Accessed August 11, 2021.

[39] Kontopantelis E , Doran T, Springate DA, et al. Regression based quasi-experimental approach when randomisation is not an option: interrupted time series analysis. *BMJ*. 2015;350:h2750. 26058820 10.1136/bmj.h2750 PMC4460815

[40] Bernal JL , Cummins S, Gasparrini A. The use of controls in interrupted time series studies of public health interventions. *Int J Epidemiol*. 2018;47(6):2082–2093. 29982445 10.1093/ije/dyy135

[41] Penfold RB , Zhang F. Use of interrupted time series analysis in evaluating health care quality improvements. *Acad Pediatr*. 2013;13(6 suppl):S38–S44. 24268083 10.1016/j.acap.2013.08.002

[42] Piccininni M , Konigorski S, Rohmann JL, et al. Directed acyclic graphs and causal thinking in clinical risk prediction modeling. *BMC Med Res Methodol*. 2020;20(1):179–179. 32615926 10.1186/s12874-020-01058-z PMC7331263

[43] Alvarado S , Cavada G, Villena R, et al. Impact of the 10-valent pneumococcal conjugate vaccine on the southern area of Santiago (Chile), 2009–2015 [in Spanish]. *Rev Panam Salud Publica*. 2018;42:e155. 31093183 10.26633/RPSP.2018.155 PMC6398315

[44] De Oliveira LH , Camacho LAB, Coutinho ESF, et al. Impact and effectiveness of 10 and 13-valent pneumococcal conjugate vaccines on hospitalization and mortality in children aged less than 5 years in Latin American countries: a systematic review. *PLoS One*. 2016;11(12):1–25. 10.1371/journal.pone.0166736 PMC5152835 27941979

[45] de Oliveira LH , Shioda K, Valenzuela MT, et al. Declines in pneumonia mortality following the introduction of pneumococcal conjugate vaccines in Latin American and Caribbean countries. *Clin Infect Dis*. 2021;73(2):306–313. 32448889 10.1093/cid/ciaa614 PMC8516507

[46] Tsaban G , Ben-Shimol S. Indirect (herd) protection, following pneumococcal conjugated vaccines introduction: a systematic review of the literature. *Vaccine*. 2017;35(22):2882–2891. 28449971 10.1016/j.vaccine.2017.04.032

[47] Carnalla-Barajas MN , Soto-Noguerón A, Sánchez-Alemán MA, et al. Changing trends in serotypes of *S. pneumoniae* isolates causing invasive and non-invasive diseases in unvaccinated population in Mexico. *Int J Infect Dis*. 2017;58:1–7. 28216181 10.1016/j.ijid.2017.02.005

[48] Pan American Health Organization . Country reports through the PAHO-WHO/UNICEF Joint Reporting Form. https://ais.paho.org/imm/IM_JRF_COVERAGE.asp. Accessed September 6, 2021.

[49] Carolan PL . Pediatric bronchitis. https://emedicine.medscape.com/article/1001332-overview#a4. Published October 11, 2019. Updated March 20, 2023. Accessed September 7, 2021.

[50] Fleming DM , Elliot AJ. The management of acute bronchitis in children. *Expert Opin Pharmacother*. 2007;8(4):415–426. 17309336 10.1517/14656566.8.4.415

[51] Tin Tin Htar M , Yerramalla MS, Moïsi JC, et al. The burden of respiratory syncytial virus in adults: a systematic review and meta-analysis. *Epidemiol Infect*. 2020;148:e48. 32052719 10.1017/S0950268820000400 PMC7078512

[52] Birkmeyer JD , Barnato A, Birkmeyer N, et al. The impact of the COVID-19 pandemic on hospital admissions in the United States. *Health Aff*. 2020;39(11):2010–2017. 10.1377/hlthaff.2020.00980 PMC7769002 32970495

[53] Guimarães RA , Policena GM, de Paula HDSC, et al. Analysis of the impact of coronavirus disease 19 on hospitalization rates for chronic non-communicable diseases in Brazil. *PLoS One*. 2022;17(3):e0265458. 35324951 10.1371/journal.pone.0265458 PMC8947087

[54] Pan American Health Organization . Vaccinate Your Family, Protect Your Community—Final Report, Technical Advsory Group (TAG) on Vaccine-Preventable Diseases, 2011. Buenos Aires, Argentina: Pan American Health Organization; 2011. https://www.paho.org/en/documents/vaccinate-your-family-protect-your-community-final-report-technical-advisory-group-tag. Accessed December 7, 2022.

