# Supplementary material for: Using LASSO Regression to Estimate the Population-Level Impact of Pneumococcal Conjugate Vaccines
Source: Am J Epidemiol. 2023 Mar 17;192(7):1166–80. doi: 10.1093/aje/kwad061 (PMC10326487; doi:10.1093/aje/kwad061)
Supplement: Web_Material_kwad061 [file web_material_kwad061.pdf]

WEB MATERIAL

Using LASSO Regression to Estimate the Population-Level Impact of Pneumococcal Conjugate Vaccines

Anabelle Wong, Sarah C. Kramer, Marco Piccininni, Jessica L. Rohmann, Tobias Kurth, Sylvie Escolano, Ulrike Grittner, and Matthieu Domenech de Cellès

Contents

Web Appendix 1. The input variables
Web Table 1
Web Appendix 2. Statistical Models
Web Appendix 3. Outcome simulation and performance assessment
Web Figures 1–3
Web Appendix 4. Using maximum entropy bootstrap to obtain 95% confidence intervals for LASSO estimates
Web Table 2
Web Figures 4–10
Web Appendix 5. Sensitivity test after removing bronchitis and bronchiolitis
Web Figure 11
Web References

WEB APPENDIX 1

The Input Variables

We simulated the outcome based on a combination of control variables, one seasonal variable, and an offset. First, we used 5 randomly selected control variables to generate the outcome, we repeated this process five times, each time randomly selecting a different set of 5 control variables. Then, we increased the number of causal control variables to 10 and again, we repeated the process five times. Web Table 1 shows the complete list of variables, where highlighted in gray are the control variables that were ever selected to simulate the outcome. In the non-causal framework, we generated the outcome using 3 control variables, and then removed these 3 causal control variables alongside the control variables that belonged to the same chapter under the *International Classification of Diseases, Tenth Revision* (ICD-10). In Web Table 1, the control variables that were ever selected to simulate the outcome in the non-causal framework are marked with "*" and the control variables that were then removed are marked with "†".

Web Table 1. The input variables in the simulation study

Variable Type	Variable Code	Description	Exclusion
Offset	ach_noj	All non-respiratory hospitalizations	J00—99, F and O chapters
Control variables	A10–B99	Non-pneumococcal infections	A40, A49, B95
	A41	Non-pneumococcal septicemia	
	B20–24	Human immunodeficiency virus infection	
	B34	Viral infections of unspecified sites	
	C00–D48*†	Neoplasm	
	D50–D89	Hematological conditions	
	E00–99†	Endocrinological and nutritional conditions, metabolic disorders	
	E10–14*†	Diabetes	
	E40–46	Malnutrition	
	G00–99	Neurological conditions	G00—04
	H00–99	Eye and ear conditions	H10, 65, 66
	I00–99	Cardiovascular conditions	
	I60–64	Stroke	
	J20–22*†	Bronchitis and bronchiolitis	
	K00–99*†	Gastrointestinal conditions	
	K35†	Appendicitis	
	K80†	Cholelithiasis	
	L00–99	Dermatological conditions	
	M00–99	Musculoskeletal conditions	
	N00–99†	Gynecological conditions	
	N39*†	Urinary tract infection	
	P00–99	Neonatal conditions	
	P05–07	Premature delivery and low birth weight	
	Q00–99	Congenital or developmental conditions	
	R00–99	Symptoms, signs, abnormal clinical or lab findings without diagnosis	
	S00–T99	Injury, poisoning, and conditions due to external causes	
	U00–99	Codes for special purposes	
	V00–Y99	Accidents and trauma	
	Z00–99*†	Health examinations and disease screening	
Seasonal variables	Cosine waves	$\cos(s * (2\pi/12) * t)$, $1 \leq s \leq 6$	
	Sine waves	$\sin(s * (2\pi/12) * t)$, $1 \leq s \leq 5$	
		(t is measured in months)	

WEB APPENDIX 2

Statistical Models

Statistical model – LASSO regression

LASSO is an extension of generalized linear regression that decreases the variance of regression coefficients and the prediction error by adding a term to the log-likelihood to penalize the complexity of the model[1]. This leads to a parsimonious model with a subset of control variables that best predicts the outcome. To estimate the penalty parameter, we first generated a grid of 100 values for the penalty parameter and fitted LASSO regression to the pre-vaccine period data for each value in the grid. Next, we selected the best value for the penalty using either 10-fold cross validation (CV) or Akaike Information Criterion (AIC)[2]. In a 10-fold CV, the pre-vaccine data period was randomly divided into 10 groups of equal size, with 9 groups forming the training set and 1 group forming the test set. A model was fit on the training set and the minimized mean squared error (MSE) was obtained when tested on the test set. This was repeated 10 times to yield an average MSE. This was repeated 100 times on each value in the grid of penalty parameter and the penalty with the lowest MSE was selected. Using the AIC for the penalty selection, we fitted LASSO regression to the pre-vaccine data period and the penalty with the lowest AIC was selected.

We tested two variants of the LASSO regression model: the first one included all seasonal variables by default (season-forced, SF); the second one treated seasonal variables as control variables and allowed LASSO regression to select from them (season-unforced, SU). The selected model was re-fitted onto the entire pre-vaccine period to predict the counterfactual outcome ($\widetilde{Y_T}$) during the evaluation period – that is, the hospitalization counts that would have occurred in the population if PCV had not been introduced, assuming the distribution and associations of the population features captured in the pre-vaccine period data remained unchanged. With the LASSO-predicted counterfactual under the no-vaccine scenario and the observed outcome (Y_T), we calculated the vaccine impact using equation 1. An IRR less than 1 indicates a reduction in all-cause pneumonia hospitalization due to the vaccination program.

$$IRR = \frac{Y_T}{\widetilde{Y_T}} = \frac{\sum_{t \in T} Y_t}{\sum_{t \in T} \widetilde{Y_t}} \tag{1}$$

where T is the set of time points during the evaluation period.

Statistical model – other methods

We compared LASSO regression to three established methods in the field of vaccine impact estimation. The key features of the implementation of LASSO regression and all comparator methods are summarized in Web Table 2. The three comparator methods are described below:

1. Interrupted Time Series (ITS) is a method that includes an indicator variable for vaccination, secular trends before and after PCV introduction, and background seasonality[3–6]. Following the procedures described in Bernal et al.[4], we applied a standard ITS with a Poisson model that contained the date, PCV's availability coded as an indicator variable (0 = no PCV, 1 = PCV in place), PCV's continuous effect coded as the time elapsed from PCV introduction, and all the seasonal variables as covariates, with the logarithm of non-respiratory hospitalization as the offset. The model was fitted to the whole period of data and the vaccine impact was calculated using equation 2:

$$IRR = \frac{\hat{Y}_T}{\widetilde{Y_T}} = \frac{\sum_{t \in T} \hat{Y}_t}{\sum_{t \in T} \widetilde{Y}_t} \qquad (2)$$

   where $\hat{Y}_T$ is the fitted outcome and $\widetilde{Y_T}$ is the counterfactual outcome during the evaluation period. We used the fitted model to predict the counterfactual outcome $\widetilde{Y_T}$ during the evaluation period in the absence of vaccination (i.e., indicator variable set to "0" for all time points).

2. In accordance with the synthetic control (SC) method[7,8], time series of different control variables were weighted according to their fit to the outcome time series in the pre-vaccine period using Bayesian variable selection. The weighted time series were jointly used to predict the counterfactual outcome $\widetilde{Y_T}$. The model was adjusted for background seasonality using 11 monthly indicator variables and the logarithm of non-respiratory hospitalization was included as a covariate. The vaccine impact was calculated using equation 1.

3. For the seasonal-trend decomposition using LOESS plus principal components analysis (STL+PCA) method, a smoothed trend for each of the control variable's time-series was extracted with seasonal-trend decomposition using locally-estimated scatterplot smoothing (LOESS)[9]. A PCA was performed on the extracted smoothed trends, and the first principal component was selected as the composite trend, which was used as a covariate in a regression model to predict the counterfactual outcome $\widetilde{Y_T}$. The vaccine impact was calculated using equation 1.

WEB APPENDIX 3

Outcome Simulation and Performance Assessment

Outcome simulation

Web Figure 1 shows the procedure for outcome simulation.

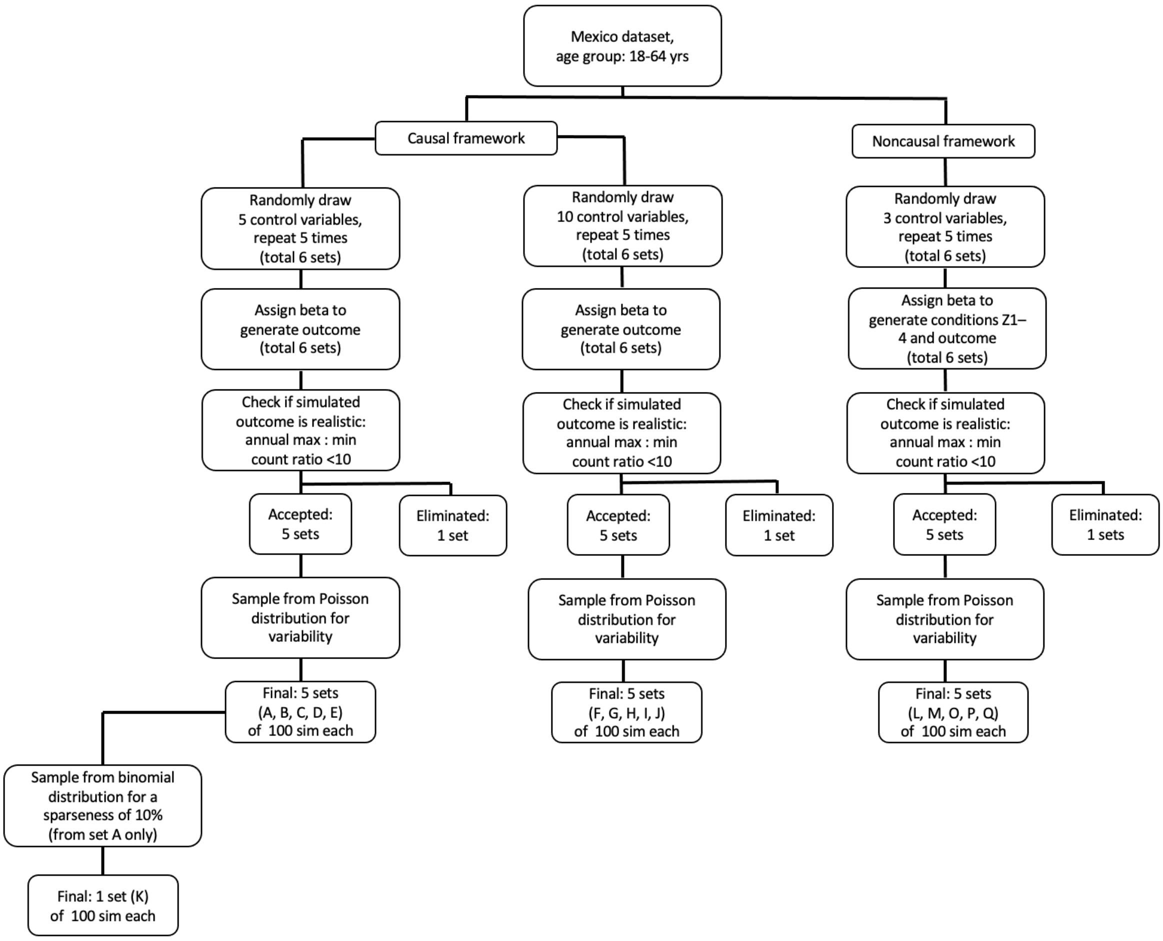

Web Figure 1. Flowchart illustrating the procedure of outcome simulation.

Performance assessment with simulated data

To assess the performance of all methods to estimate vaccine impact, we designed a simulation study. We generated the outcome, monthly pneumonia hospitalization (Y_t), based on a combination of n (n = *{5, 10}*) control variables ($X_1, X_2, \ldots, X_n$) randomly selected from the list of control variables available in the Mexico data set[7]. We then incorporated an intercept (α), the logarithm of non-respiratory hospitalization as the offset ($\ln(NRH_t)$), background seasonality (S_t), and a vaccine impact component (γ) into the equation to generate the logarithm of the expected number of monthly pneumonia hospitalization. A pre-determined value was assigned

to γ starting from the time point of PCV introduction (t_{vac}). Assuming a Poisson distribution for the outcome, we simulated 100 time series; thus, the variability of the simulated time series originated from the Poisson variation. The model is represented by equation 2:

$$Y_t \sim \text{Poisson}(\mu_t) \tag{2}$$

$$\ln(\mu_t) = \alpha + \ln(NRH_t) + \sum_{i=1}^{n} \beta_i X_{it} + S_t + \gamma \, \mathbb{1}(t \geq t_{vac})$$

$$where \ \alpha = \ln\left(\frac{\bar{Y}}{\overline{NRH}}\right)$$

$$and \ S_t = \sum_{s=1}^{6} \delta_s \cos\left(\frac{2\pi st}{12}\right) + \sum_{s=1}^{5} \zeta_s \sin\left(\frac{2\pi st}{12}\right)$$

The intercept, α, was calculated as the logarithm of the mean ratio of pneumonia hospitalization to all non-respiratory hospitalization ($\ln(\bar{Y}/\,\overline{NRH})$). For the association of any control variable and the outcome not to be unrealistically strong, the values assigned to the β of the included control variables were randomly sampled from a uniform distribution with range –0.3 to 0.3, such that a change of one standard-deviation in the control variable, holding the other variables constant, would result in 0.74- to 1.35-fold change in the outcome. S_t was modelled as a Fourier series of 11 terms that consisted of 6 cosine and 5 sine functions. We assigned a value of 0.5 to the δ of the first cosine function such that the outcome peaked in January and oscillated approximately 50% above and below the annual mean to mimic pneumonia seasonality in the real world. The impact of vaccination was modeled by the parameter γ. In all simulations, we assumed a vaccine with null impact ($\gamma = 0$), IRR=1, except in the sensitivity analysis where 1 – IRR = 10%. The simulated data were screened to ensure they were realistic, such that the maximum ratio of annual maximum-to-minimum for the expected count of the outcome in any simulation set would not exceed 10.

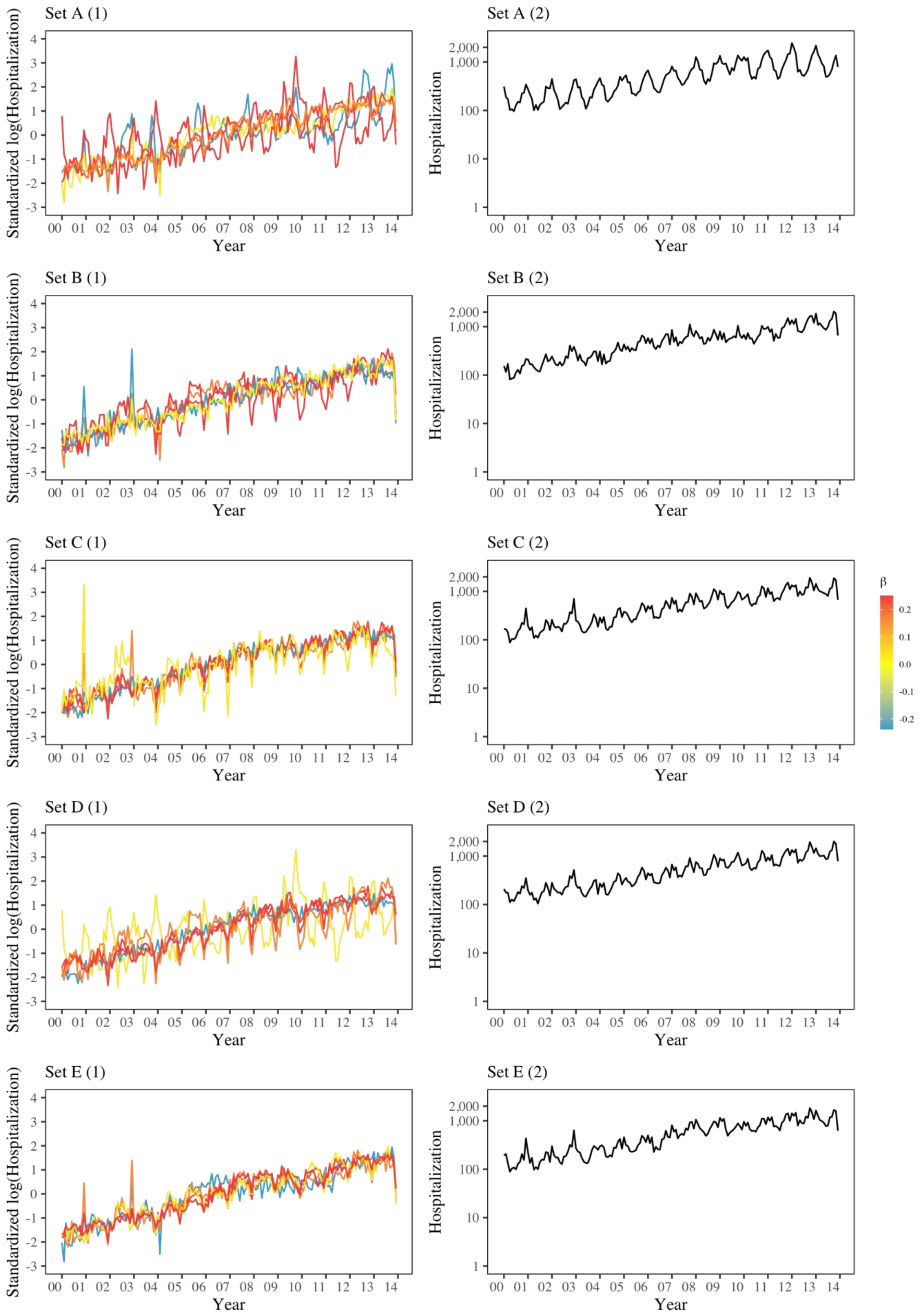

Web Figure 2. Time series of (1) the five control variables (log-transformed, standardized) selected for outcome simulation and (2) the simulated outcome in sets A to E.

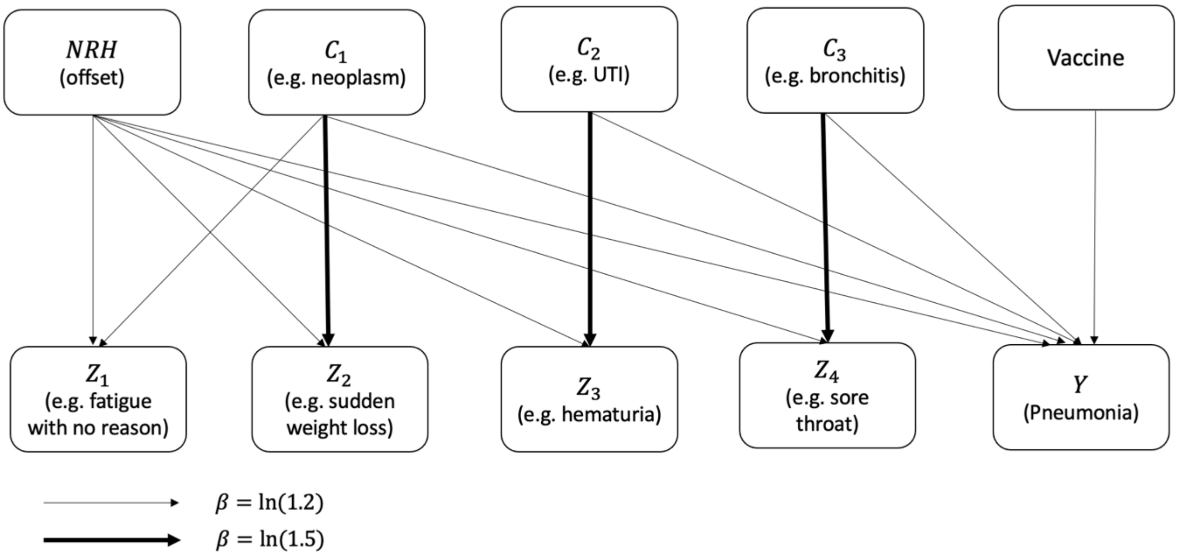

Web Figure 3. Directed Acyclic Graph (DAG) for outcome simulation under the non-causal framework.

The causal relationships represented in this DAG depict the relationships between the control variables (Z_1, Z_2, Z_3, Z_4) and the outcome (Y) simulated under the non-causal framework in this study. The thickness of the arrow represented the magnitude of beta coefficient assigned to the causes (C_1, C_2, C_3). The causes (marked with "*" in Web Table 1) together with their associated control variables (marked with "†" in Web Table 1) were then removed from the list of control variables for model testing.

WEB APPENDIX 4

Using Maximum Entropy Bootstrap to Obtain 95% Confidence Intervals for Lasso Estimates

The ways to obtain different 95% uncertainty intervals (UI) for different methods in the application to real-world data are summarized in Web Table 2.

Web Table 2. A summary of how 95% uncertainty intervals of estimates were obtained for different methods in the application to real-world data.

Method	Uncertainty Intervals (UI)	How the UI were obtained	Type of uncertainty being measured	Figures
**LASSO**	Prediction intervals (PI)	Extracted from the 2.5th & 97.5th percentiles of the Poisson distribution of the predicted counterfactual	Distribution uncertainty	Fig. 3, Web Fig. 10
**LASSO**	Confidence intervals (CI)	Extracted from the 2.5th & 97.5th percentiles of the IRR estimate from 100 maximum entropy bootstrap samples	Parametric uncertainty	Web Fig. 10
**SC**	Credible intervals (CrI)	Extracted from the 2.5th & 97.5th percentiles of the Bayesian posterior distributions	Parametric uncertainty	Fig. 3, Web Fig. 10

To calculate approximate 95% CI of the IRR for LASSO, we explored a non-parametric bootstrap approach. Specifically, we used the maximum entropy algorithm (implemented in the R package "meboot"[10]) to construct bootstrap replicates of the log-transformed time series for the outcome, the offset, and every control variable. We then converted the bootstrapped time series back to the natural scale, and assembled them to form bootstrap replicates of the original data set. We then fit LASSO-SF and estimated the IRR for each bootstrap data set, and calculated the 95% CI based on the 2.5th and 97.5th percentiles of the resulting IRR distribution. The results, presented in Web Figure 10, were based on a sample of 100 bootstrapped data sets.

Web Figure 4. Seasonal and control variables selected by LASSO-SU for simulation 1 to 100 in simulation sets A to E (5 causal control variables).

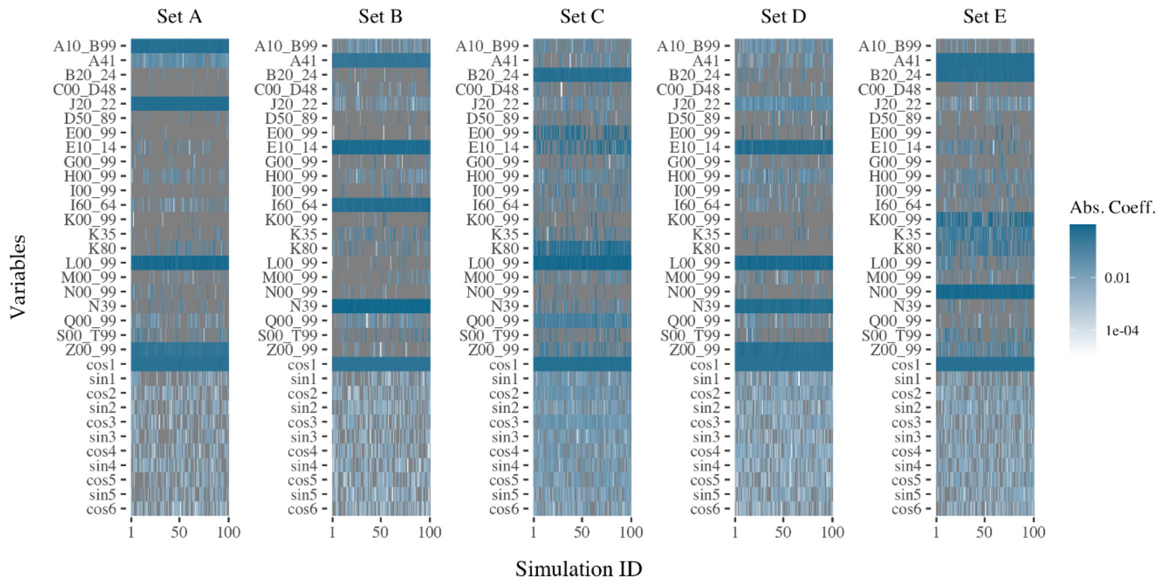

Each panel represents a scenario with outcome simulated with a different set of five causal control variables (which remained in data set) and one seasonal variable. The five causal control variables used for the simulation set from left to right are: set A – health exams (Z00–99), bronchitis and bronchiolitis (J20–22), dermatological condition (L00–99), non-pneumonia infection (A10–B99), and non-pneumococcal septicemia (A41); set B – non-pneumococcal septicemia (A41), urinary tract infection (UTI, N39), diabetes (E10–14), stroke (I60–64), and injury (S00–T99); set C – human immunodeficiency virus (HIV) infection (B20–24), cholelithiasis (K80), dermatological condition (L00–99), endocrinological condition (E00–99), and congenital condition (Q00–99); set D – UTI (N39), dermatological conditions (L00–99), health exams (Z00–99), diabetes (E10–14), and bronchitis and bronchiolitis (J20–22); and set E –HIV infection (B20–24), gynecological conditions (N00–99), gastrointestinal conditions (K00–99), non-pneumococcal septicemia (A41), and appendicitis (K35). In each panel for simulation 1 to 100, the absolute value of the coefficient estimated for the variable is represented by shades of blue and non-selection by LASSO-SU is represented by gray.

Web Figure 5. The difference in cross-validation (CV) vs. Akaike Information Criterion (AIC) model selection in LASSO-SF and LASSO-SU.

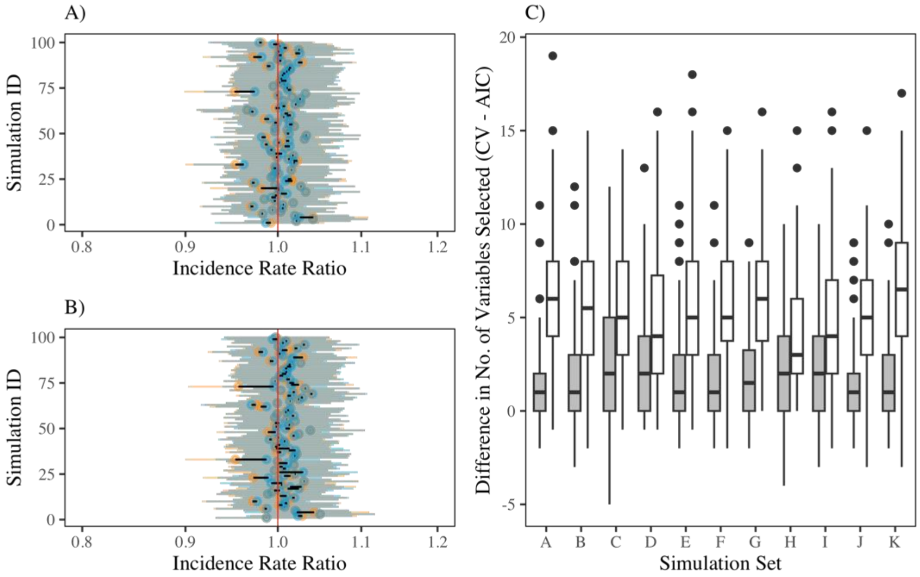

The point estimates and 95% uncertainty intervals were similar using CV (orange) or AIC (blue) to select model, in both LASSO method variants, A) LASSO-SF and B) LASSO-SU. C) Selection using CV resulted in models with more variables compared to using AIC across simulation sets.

Web Figure 6. Sensitivity test results from LASSO-SF and LASSO-SU on simulation sets A to E (5 causal control variables) with a non-null, low impact vaccine (1-IRR = 10%).

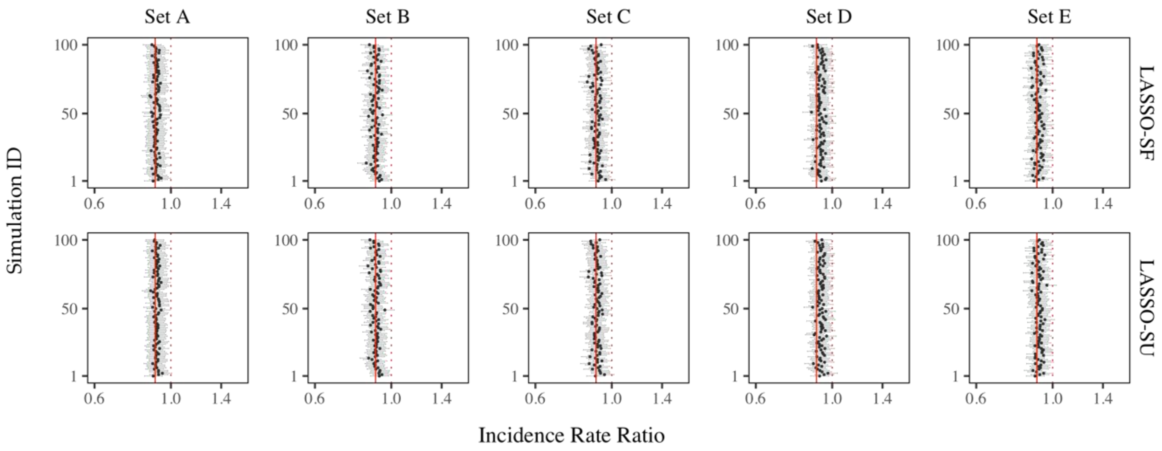

Each row shows the estimates using a different method, from top to bottom: LASSO-SF, LASSO-SU. Each column represents a scenario with outcome simulated with a different set of five causal control variables (which remained in data set) and one seasonal variable; the five causal control variables used for the simulation set from left to right are: set A – health exams, bronchitis and bronchiolitis, dermatological condition, non-pneumonia infection, and non-pneumococcal septicemia; set B – non-pneumococcal septicemia, urinary tract infection (UTI), diabetes, stroke, and injury; set C – human immunodeficiency virus (HIV) infection, cholelithiasis, dermatological condition, endocrinological condition, congenital condition; set D – UTI, dermatological conditions, health exams, diabetes, and bronchitis and bronchiolitis; and set E – HIV infection, gynecological conditions, gastrointestinal conditions, non-pneumococcal septicemia, and appendicitis. Each panel shows the result in 100 simulations, the points represent the estimated Incidence Rate Ratio (IRR) and the error bars represent the 95% uncertainty intervals. The red vertical solid line indicates the IRR corresponding to the true vaccine impact (10% impact) in the simulation (i.e., IRR = 0.9); the red vertical dotted line indicates the IRR corresponding to null impact (i.e., IRR = 1).

Web Figure 7. Incidence rate ratios estimated by various methods for simulation 1 to 100 in simulation sets F to K (F–J: 10 causal control variables, K: sparse outcome).

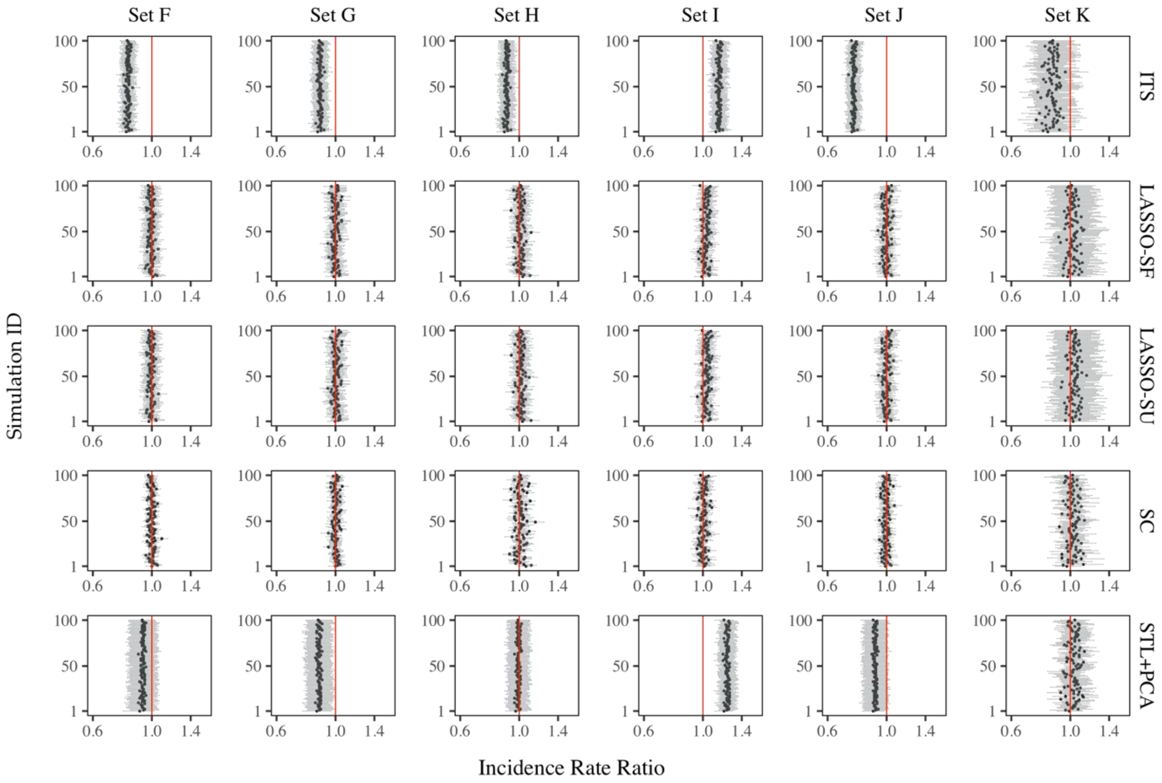

Each row shows the estimates using a different method, from top to bottom: ITS, LASSO-SF, LASSO-SU, SC, and STL+PCA. Each column represents a scenario with outcome simulated with a different set of ten causal control variables (which remained in data set) and one seasonal variable; the ten causal control variables used for the simulation set from left to right are: set F – health exams, bronchitis and bronchiolitis, dermatological condition, non-pneumonia infection, non-pneumococcal septicemia, hematological condition, cardiovascular condition, neoplasm, cholelithiasis, and injury; set G – non-pneumococcal septicemia, urinary tract infection (UTI), diabetes, stroke, injury, human immunodeficiency virus (HIV) infection, neurological condition, neoplasm, hematological condition, and bronchitis and bronchiolitis; set H – HIV infection, cholelithiasis, dermatological condition, endocrinological condition, congenital condition, cardiovascular condition, hematological condition, appendicitis, neoplasm, and stroke; set I – UTI, dermatological conditions, health exams, diabetes, bronchitis and bronchiolitis, cholelithiasis, cardiovascular condition, congenital condition, musculoskeletal condition, and neurological condition; set J – HIV infection, gynecological conditions, gastrointestinal conditions, non-pneumococcal septicemia, appendicitis, hematological condition, congenital

condition, stroke, musculoskeletal condition, and cholelithiasis; and set K – same as set A but outcome count reduced to 10% as sparse. Each panel shows the result in 100 simulations, the points represent the estimated Incidence Rate Ratio (IRR) and the error bars represent the 95% uncertainty intervals. The red vertical line indicates the true vaccine impact in the simulation, which is 1 in all of our simulation scenarios; here, an IRR larger than 1 means underestimation of vaccine impact and an IRR lower than 1 means overestimation of vaccine impact.

Web Figure 8. Variables selected by LASSO-SU in simulation sets F to J (10 causal control variables).

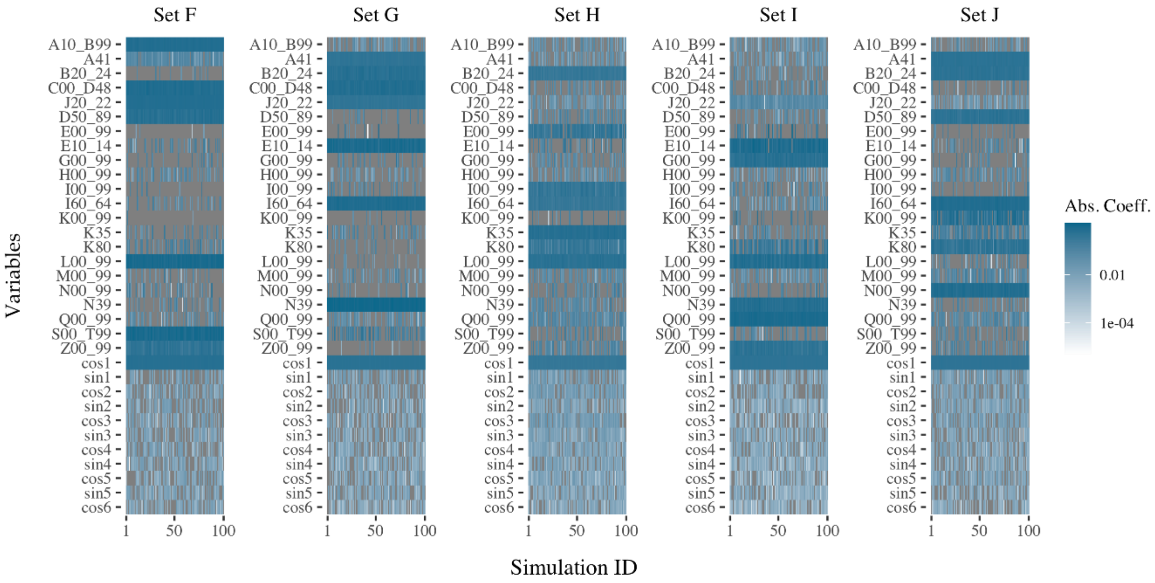

Each panel represents a scenario with outcome simulated with a different set of five causal control variables (which remained in data set) and one seasonal variable. The five causal control variables used for the simulation set from left to right are: set F – health exams (Z00–99), bronchitis and bronchiolitis (J20–22), dermatological condition (L00–99), non-pneumonia infection (A10–B99), non-pneumococcal septicemia (A41); hematological condition (D50–89), cardiovascular condition (I00–99), neoplasm (C00–D48), cholelithiasis (K80), and injury (S00–T99); set G – non-pneumococcal septicemia (A41), urinary tract infection (UTI, N39), diabetes (E10–14), stroke (I60–64), injury (S00–T99), human immunodeficiency virus (HIV) infection (B20–24), neurological condition (G00–99), neoplasm (C00–D48), hematological condition (D50–89), and bronchitis and bronchiolitis (J20–22); set H – HIV infection (B20–24), cholelithiasis (K80), dermatological condition (L00–99), endocrinological condition (E00–99), congenital condition (Q00–99), cardiovascular condition (I00–99), hematological condition (D50–89), appendicitis (K35), neoplasm (C00–D48), and stroke (I60–64); set I – UTI (N39), dermatological conditions (L00–99), health exams (Z00–99), diabetes (E10–14), bronchitis and bronchiolitis (J20–22), cholelithiasis (K80), cardiovascular condition (I00–99), congenital condition (Q00–99), musculoskeletal condition (M00–99), and neurological condition (G00–99); and set J – HIV infection (B20–24), gynecological conditions (N00–99), gastrointestinal conditions (K00–99), non-pneumococcal septicemia (A41), appendicitis (K35), hematological condition (D50–89), congenital condition (Q00–99), stroke (I60–64), musculoskeletal condition (M00–99), and cholelithiasis (K80). In each panel for simulation 1 to 100, the absolute value of the coefficient estimated for the variable is represented by shades of blue and non-selection by LASSO-SU is represented by gray.

Web Figure 9. Seasonal and control variables selected by LASSO-SU in for simulation 1 to 100 in simulation sets L to P.

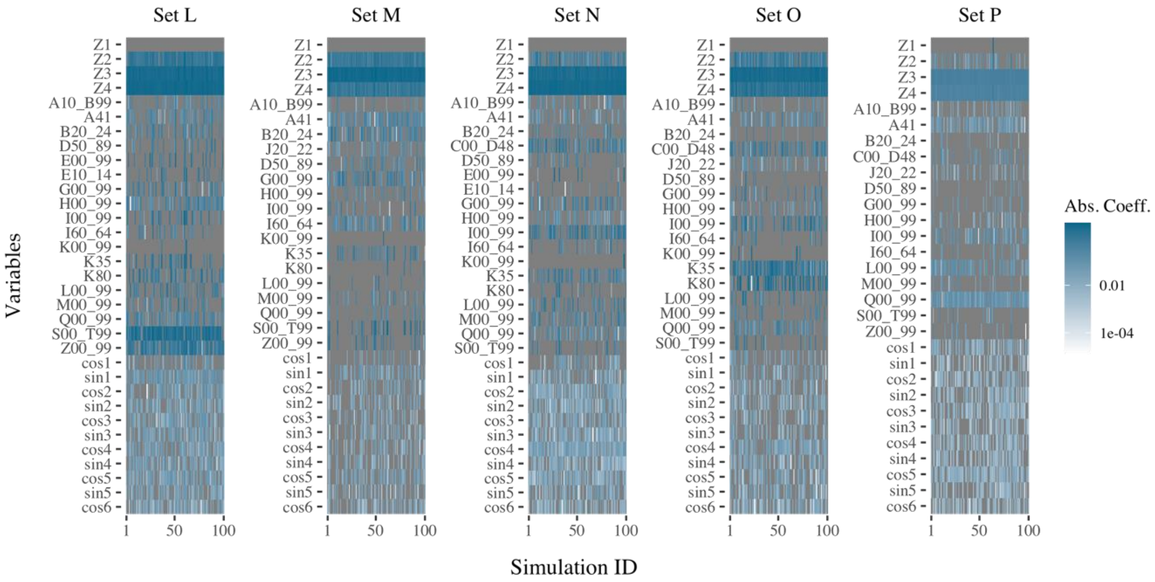

Each panel represents a scenario with outcome simulated from a different set of three causal control variables (which were then removed alongside other control variables under the same ICD chapter, leaving behind only non-causal control variables) and one seasonal variable. The three causal control variables used for the simulation set from left to right are: set L – neoplasm (C00–D48), urinary tract infection (UTI, N39), and bronchitis and bronchiolitis (J20–22); set M – neoplasm (C00–D48), UTI (N39), and diabetes (E10–14); set N – health exams (Z00–99), UTI (N39), and bronchitis and bronchiolitis (J20–22); set O – health exams (Z00–99), UTI (N39), and diabetes (E10–14); and set P – gastrointestinal conditions (K00–99), UTI (N39), and diabetes (E10–14). In each panel for simulation 1 to 100, the absolute value of the coefficient estimated for the variable is represented by shades of blue and non-selection by LASSO-SU is represented by gray.

Web Figure 10. Age-group-specific incidence rate ratios (IRR) for four countries, estimated by LASSO-SF (with different uncertainty intervals) and SC.

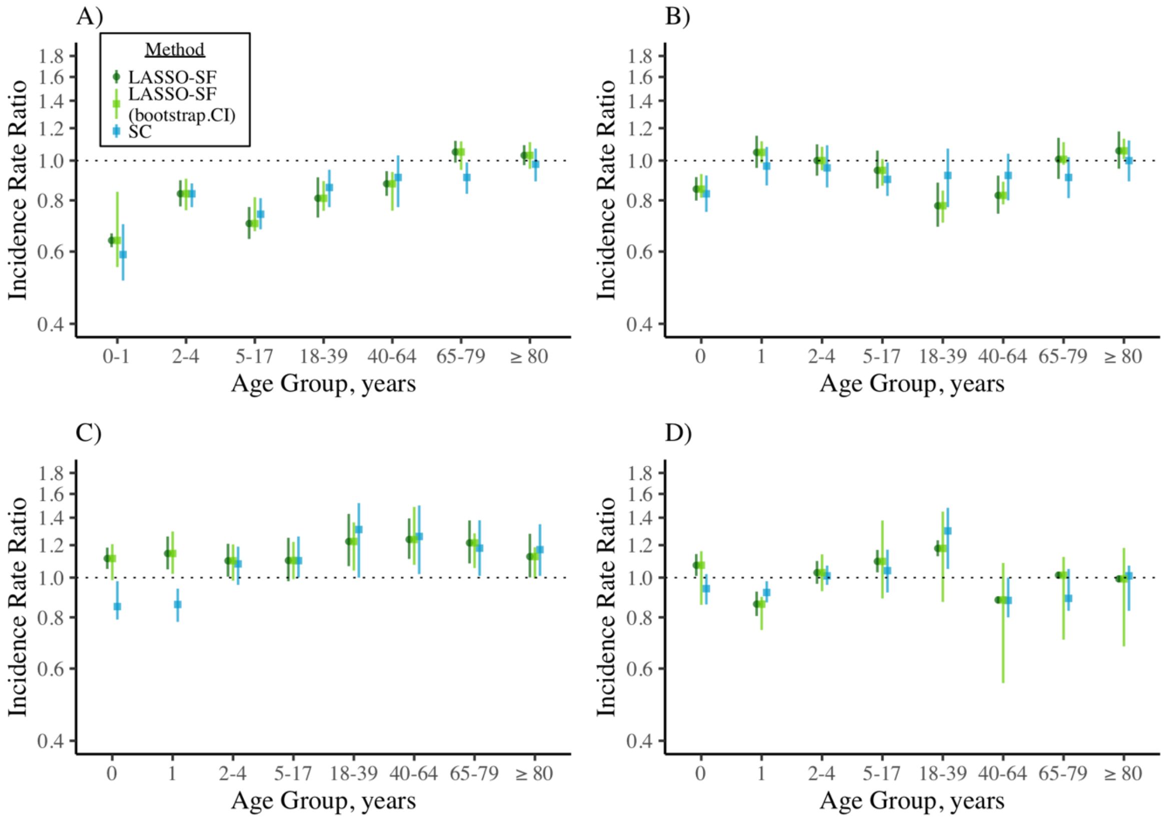

Each panel shows the age-group-specific IRR for all-cause pneumonia in a population whose infants were vaccinated with pneumococcal conjugate vaccines (PCV) compared to a counterfactual population in which PCV was never introduced, estimated by LASSO-SF (dark green circle: 95% prediction interval from Poisson distribution; light green square: 95% confidence interval from maximum entropy bootstrapping[10] of the outcome and of the covariates) and SC (blue square: 95% credible intervals). The four countries are A) Chile; B) Ecuador; C) Mexico; and D) the US.

WEB APPENDIX 5

Sensitivity Test After Removing Bronchitis and Bronchiolitis

As a sensitivity test, we removed “bronchitis and bronchiolitis” from the list of control variables that LASSO regression and SC could choose from and re-analyzed the pneumonia hospitalization data from Chile, Ecuador, Mexico and the US. In Chile, the results remained similar. In Ecuador, the reduction in all-cause pneumonia hospitalization detected in the main analysis (Figure 4, panel B) attenuated in the youngest age group, but remained statistically significant in age groups 18 to 64 years (Web Figure 11, panel B). In Mexico, the reduction in the youngest two age groups detected by SC in the main analysis (Figure 4, panel C) was also attenuated and was no longer statistically significant (Web Figure 11, panel C). In the US, only a marginal reduction was detected by LASSO-SF and LASSO-SU in the age group 40 to 64 years and not in older adults before removing “bronchitis and bronchiolitis” (Figure 4, panel D), but after doing so, a more pronounced reduction was observed in all the age groups from 18 to 79 years (Web Figure 11, panel D).

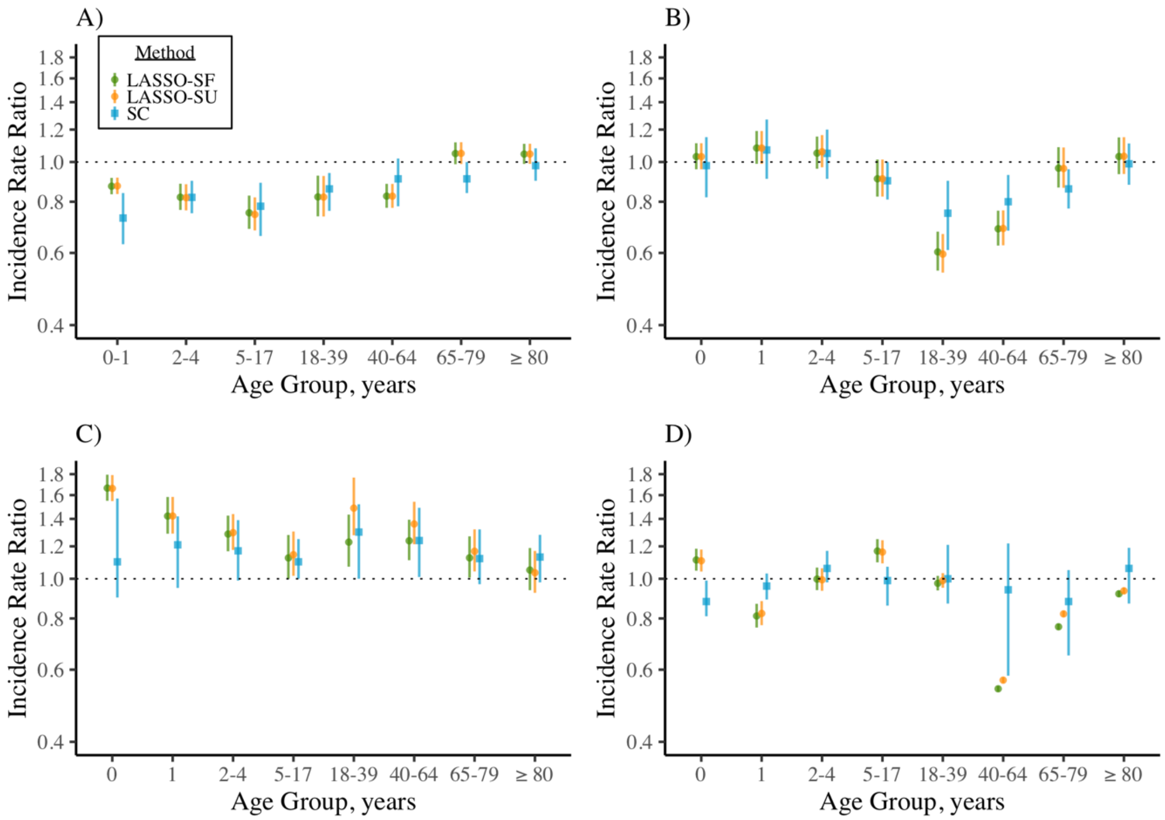

Web Figure 11. Age-group-specific incidence risk ratios (IRR) for all-cause pneumonia in four countries after removing “bronchitis and bronchiolitis”, estimated by two LASSO methods and SC.

Each panel shows the age-group-specific IRR for all-cause pneumonia in a population whose infants were vaccinated with pneumococcal conjugate vaccines (PCV) compared to a counterfactual population in which PCV was never introduced, estimated by LASSO-SF (green), LASSO-SU (orange), and SC (blue). The four countries are A) Chile; B) Ecuador; C) Mexico; and D) the US. The 95% prediction intervals (PI) of estimates by LASSO-SF and LASSO-SU are shown by the error bars joined at a circle; the 95% credible intervals (CrI) of estimates by SC are shown by the error bars joined at a square. 95% PI and 95% CrI are different uncertainty measures and are thus not directly comparable.

Web References

1. Tibshirani R. Regression Shrinkage and Selection Via the Lasso. *J R Stat Soc Ser B*. 1996;58(1):267-288.
2. James G, Witten D, Hastie T, Tibshirani R. *An Introduction to Statistical Learning*. New York: Springer; 2013.
3. Lecy J, Fusi F. Interrupted Time Series. Published 2020. https://ds4ps.org/pe4ps-textbook/docs/p-020-time-series.html (Accessed August 11, 2021)
4. Bernal JL, Cummins S, Gasparrini A. Interrupted time series regression for the evaluation of public health interventions: A tutorial. *Int J Epidemiol*. 2017;46(1):348-355. doi:10.1093/ije/dyw098
5. Grijalva CG, Nuorti JP, Arbogast PG, Martin SW, Edwards KM, Griffin MR. Decline in pneumonia admissions after routine childhood immunisation with pneumococcal conjugate vaccine in the USA: a time-series analysis. *Lancet*. 2007;369(9568):1179-1186. doi:10.1016/S0140-6736(07)60564-9
6. Ouldali N, Levy C, Minodier P, et al. Long-term Association of 13-Valent Pneumococcal Conjugate Vaccine Implementation with Rates of Community-Acquired Pneumonia in Children. *JAMA Pediatr*. 2019;173(4):362-370. doi:10.1001/jamapediatrics.2018.5273
7. Bruhn CAW, Hetterich S, Schuck-Paim C, et al. Estimating the population-level impact of vaccines using synthetic controls. *Proc Natl Acad Sci U S A*. 2017;114(7):1524-1529. doi:10.1073/pnas.1612833114
8. Kleynhans J, Tempia S, Shioda K, von Gottberg A, Weinberger DM, Cohen C. Estimated impact of the pneumococcal conjugate vaccine on pneumonia mortality in South Africa, 1999 through 2016: An ecological modelling study. *PLoS Med*. 2021;18(2):1-15. doi:10.1371/JOURNAL.PMED.1003537
9. Shioda K, Schuck-Paim C, Taylor RJ, et al. Challenges in Estimating the Impact of Vaccination with Sparse Data. *Epidemiology*. 2019;30(1):61-68. doi:10.1097/EDE.0000000000000938
10. Vinod HD, López-de-Lacalle J. Maximum entropy bootstrap for time series: The meboot R package. *J Stat Softw*. 2009;29(5):1-19. doi:10.18637/jss.v029.i05
